# Mechanism of activation and regulation of deubiquitinase activity in MINDY1 and MINDY2

**DOI:** 10.1016/j.molcel.2021.08.024

**Published:** 2021-10-21

**Authors:** Syed Arif Abdul Rehman, Lee A. Armstrong, Sven M. Lange, Yosua Adi Kristariyanto, Tobias W. Gräwert, Axel Knebel, Dmitri I. Svergun, Yogesh Kulathu

**Affiliations:** 1 MRC Protein Phosphorylation & Ubiquitylation Unit, School of Life Sciences, University of Dundee, Dow Street, Dundee DD1 5EH, UK; 2 European Molecular Biology Laboratory, Hamburg Unit, EMBL c/o DESY, Notkestr. 85, 22607 Hamburg, Germany

**Keywords:** ubiquitylation, deubiquitinase, crystal structure, polyubiquitin, protease, enzyme mechanism, protein degradation, proteasome, conformational change, autoinhibition

## Abstract

Of the eight distinct polyubiquitin (polyUb) linkages that can be assembled, the roles of K48-linked polyUb (K48-polyUb) are the most established, with K48-polyUb modified proteins being targeted for degradation. MINDY1 and MINDY2 are members of the MINDY family of deubiquitinases (DUBs) that have exquisite specificity for cleaving K48-polyUb, yet we have a poor understanding of their catalytic mechanism. Here, we analyze the crystal structures of MINDY1 and MINDY2 alone and in complex with monoUb, di-, and penta-K48-polyUb, identifying 5 distinct Ub binding sites in the catalytic domain that explain how these DUBs sense both Ub chain length and linkage type to cleave K48-polyUb chains. The activity of MINDY1/2 is inhibited by the Cys-loop, and we find that substrate interaction relieves autoinhibition to activate these DUBs. We also find that MINDY1/2 use a non-canonical catalytic triad composed of Cys-His-Thr. Our findings highlight multiple layers of regulation modulating DUB activity in MINDY1 and MINDY2.

## Highlights

• The catalytic domain of MINDY1/2 has five distinct Ub binding sites
• Dynamics of the Cys loop regulate DUB activity
• Non-canonical catalytic triad composed of Cys-His-Thr
• MINDY1/2 uses an exo- or endo-cleavage mode that is determined by Ub chain length

## Graphical abstract

**Figure:**
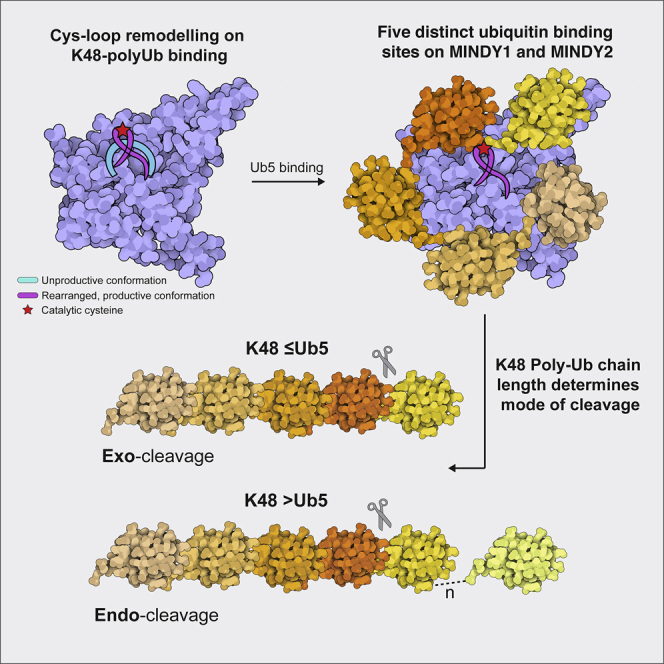

## Teaser

Abdul Rehman et al. uncover multiple layers of regulation of MINDY1 and MINDY2, two recently discovered K48-specific deubiquitinases. They report that MINDY1/2 uses a non-canonical catalytic mechanism, and its catalytic domain has five distinct ubiquitin binding sites, which establishes a polyUb length-dependent cleavage mode.

## Introduction

Nearly all aspects of eukaryotic cell biology are influenced by the posttranslational modification (PTM) of proteins with ubiquitin (Ub). Typically, Ub is tagged onto a substrate protein by the formation of an isopeptide bond between its C-terminal carboxyl group and the ε-amine group of a lysine residue on the substrate. This primary Ub can be extended where 1 of its 7 lysine residues (K6, K11, K27, K29, K33, K48, K63) or its N-terminal methionine (M1) can serve as an attachment point for another Ub to result in the formation of polyUb chains (Komander and Rape, 2012). K48-linked ubiquitylation, the most prevalent linkage type, targets modified proteins for destruction by the proteasome, thereby maintaining a functional proteome, the failure of which is the underlying cause for many diseases, including neurodegenerative disorders (Dikic, 2017; Hershko and Ciechanover, 1998).

Given their wide-ranging effects on cell signaling and eukaryotic biology, ubiquitylation is regulated by dedicated Ub proteases called deubiquitinases (DUBs), which can remove Ub from the substrate or trim polyUb chains (Clague et al., 2019; Leznicki and Kulathu, 2017). Of the 100 DUBs known so far in humans, the majority of them show no linkage preference and can hydrolyze all polyUb chain types, whereas few DUBs exhibit exquisite linkage selectivity and cleave only specific linkage types (Abdul Rehman et al., 2016; Faesen et al., 2011; Kwasna et al., 2018; Mevissen et al., 2013). The mode of Ub recognition by the DUB determines whether it is linkage specific. For instance, the USP family DUBs have a large S1 Ub binding site and their activity depends on distal Ub binding. However, some DUBs rely on additional proximal Ub interactions at the S1′ site to orient a specific lysine or the N-terminal methionine toward the catalytic site, thus making them selective at cleaving specific linkages (Clague et al., 2019). A key objective of these interactions between DUBs and Ub molecules is to stabilize the linkage between Ub moieties of a chain within the active site for efficient cleavage, especially since Ub chains of different linkage types adopt distinct conformations. For instance, M1-, K33-, and K63-linked chains can exist in an open extended conformation with accessible I44 binding patches that can be recognized by DUBs (Kristariyanto et al., 2015; Ronau et al., 2016). Other linkage types, such as K6-, K11-, and K48-linked chains, adopt compact conformations and must undergo significant conformational changes to be recognized by a DUB (Békés et al., 2016; Gersch et al., 2017; Mevissen et al., 2016; Sato et al., 2017; Ye et al., 2012). Despite it being the most abundant linkage type, we do not know how K48-linked polyUb is recognized by DUBs, as there are no available structures of DUBs bound to K48-linked chains.

DUBs process Ub chains in diverse ways to modulate ubiquitylation, and the mode of chain cleavage is a factor that can influence the duration of the Ub signal. For instance, DUBs can cleave from one end of the chain (exo-DUB) to trim the Ub chain (Lee et al., 2011; Leznicki and Kulathu, 2017; Wilkinson et al., 1995). Alternatively, DUBs can hydrolyze linkages at any position within the Ub chains (endo-DUB) and thereby rapidly terminate a Ub signal. The endo-cleavage mode is characteristic of DUBs such as CYLD and A20, which regulate immune signaling (Komander et al., 2008; Lin et al., 2008). DUBs are also subject to multiple layers of regulation that ensure activity only at precise times and locations. Several DUBs exist in an autoinhibited conformation typified by a misaligned catalytic triad or occluded substrate-binding site and depend on PTMs, allosteric regulation, or substrate interactions for activation (Sahtoe and Sixma, 2015).

We recently reported the discovery of the MINDY (MIU containing novel DUB family) enzymes as a separate class of cysteine protease DUBs. DUBs of the MINDY family are evolutionarily conserved and remarkably specific at cleaving K48-linked chains (Abdul Rehman et al., 2016). MINDY1 and MINDY2 show high sequence similarity and similar domain architectures. We found that MINDY1 is an exo-DUB with a preference for cleaving long K48-linked polyUb chains. Furthermore, MINDY1 exists in an inhibited conformation and the identity of the catalytic triad is unclear. Hence, the catalytic mechanism and how MINDY1 becomes activated and specifically cleaves K48-linked Ub chains in this unique fashion are unknown. To address these questions, we determined here the crystal structures of MINDY1 and MINDY2 in complex with K48-diUb (K48-Ub_2_) and MINDY2 in complex with K48-pentaUb (K48-Ub_5_). Our structural analyses, coupled with mutational studies, reveal the mechanism of autoinhibition and activation of MINDY1 and MINDY2.

## Results

### Structure of MINDY1 in complex with K48-linked diUb

**Figure 1:**
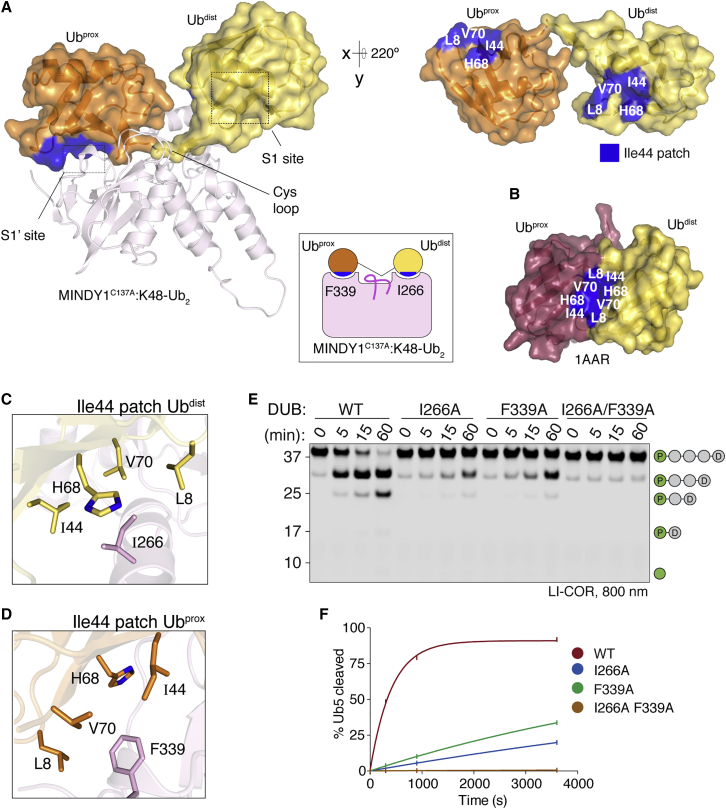
Crystal structures of MINDY1 in complex with K48-linked di-ubiquitin (A) The MINDY1^C137A^:K48-Ub_2_ complex crystal structure is shown with MINDY1 in illustration (light pink). Ub molecules are depicted with transparent surfaces (tv-orange:Ub^prox^ and yelloworange:Ub^dist^). I44 patches on Ub are colored blue, and an alternate view of the bound diUb rotated by 220° along the x axis is shown on the right side. Schematic representation of MINDY1^C137A^:K48-Ub_2_ complex (inset). (B) Surface representation of the closed conformation of K48-Ub_2_ (PDB: 1AAR) with I44 patches highlighted in blue. (C and D) Close-up views of the key residues on the MINDY1 S1 and S1′ sites and their interactions with the I44 patches on Ub^dist^ and Ub^prox^. (E) DUB assay monitoring cleavage of K48-linked pentaUb, in which Ub^prox^ is fluorescently labeled by MINDY1 and indicated mutants. (F) Quantification of pentaUb hydrolysis shown in (D). The percentage of the total intensities of Ub4, Ub3, Ub2, and Ub1 formed is shown on the y axis. n = 2; mean ± SD. See also Figures S1 and S2.

The minimal catalytic domains of MINDY1 (MINDY1^cat^) and MINDY2 (MINDY2^cat^) contain all of the specificity determinants to selectively cleave K48-linked chains (Figures S1A and S1B; Abdul Rehman et al., 2016). To understand the structural basis for the specific cleavage of K48-linked chains by MINDY1, we determined the crystal structures of catalytically dead mutants of MINDY1^cat^ and MINDY2^cat^ bound to K48-linked diUb, MINDY1^C137A^:K48-Ub_2_, and MINDY2^C266A^:K48-Ub_2_, respectively (Figures 1A and S1D; Table S4). The crystal structures of MINDY2^cat^ and MINDY1^cat^ closely resemble one another (root-mean-square deviation [RMSD] 1.05 Å), and both DUBs bind to K48 chains in a similar way (Figure S1C). Overall, we found MINDY1 and MINDY2 to use analogous mechanisms and, to simplify our description, we focus on MINDY1, highlighting any differences observed with MINDY2.

The crystal structure of MINDY1^C137A^:K48-Ub_2_ complex reveals how MINDY1^cat^ has extensive interactions with both the proximal Ub (Ub^prox^) and distal Ub (Ub^dist^) to precisely position the scissile bond across the catalytic site (Figures 1A and S1D–S1F). Ub^dist^ binds to the S1 site in MINDY1 with a buried surface area of only ∼750 Å^2^, a binding interface much smaller than those of other DUBs such as HAUSP/USP7 (1,700 Å^2^), USP21 (1,700 Å^2^), or USP2 (1,900 Å^2^), which rely more on Ub^dist^ binding for activity (Hu et al., 2002; Renatus et al., 2006; Ye et al., 2011). In the crystal structure of MINDY1 in complex with propargylated Ub (MINDY1∼Ub), a covalent complex representing the product intermediate state, the Ub occupying the S1 site exists in two alternate conformations suggestive of weak binding. One of these conformers, conformer A, corresponds to Ub^dist^ in the K48-Ub_2_ complex (Figure S2A). This suggests that Ub^prox^ interactions with MINDY1^cat^ stabilizes Ub^dist^ binding. Indeed, Ub^prox^ has a slightly larger binding interface compared to Ub^dist^ and binds with a buried surface area of ∼965 Å^2^.

When bound to MINDY1/2, the K48-linked chain adopts an extended conformation that lacks interchain Ub-Ub interactions (Figures 1A and S1D). This contrasts with the compact conformation observed for K48-linked chains in isolation, in which the I44 patches of both Ub^dist^ and Ub^prox^ form a hydrophobic interface (Figure 1B). In the extended, DUB-bound conformation, the I44 patches of both Ub^prox^ and Ub^dist^ are now engaged in interactions with the catalytic domain instead (Figure 1A). The I44 patch on Ub^dist^ primarily contacts I266 on MINDY1 (Figure 1C), and further interactions with Ub^dist^ are mediated by V210, W240, Y258, and F315, which form a binding pocket for L73 of Ub^dist^. Polar interactions between D209 and E263 in MINDY1 with R74 and R72 of Ub^dist^ further contribute to binding (Figures S2B and S2D). The I44 patch of Ub^prox^ mainly contacts F339 on MINDY1 (Figure 1D). The binding of Ub^prox^ at the S1′ site is further supported by additional cation-π interactions between Ub^prox^ F45 and MINDY1 R316, hydrogen bonds between the side chains of N317 and Ub^prox^ N60 and the backbone of Y59, and ionic interactions between Ub^prox^ R42 and MINDY1 D336 (Figures S2D–S2F).

Disruption of the binding of Ub^dist^ or Ub^prox^ in the MINDY1 mutants I266A (S1 site) and F339A (S1′ site), respectively, results in reduced activity relative to wild type (WT), as monitored by the cleavage of fluorescently labeled K48-Ub_5_ (Figures 1E and S1G). As the double mutant (I266A/F339A) completely abolishes DUB activity (Figures 1E and 1F) and residues involved in Ub^prox^ and Ub^dist^ recognition are evolutionarily conserved (Figures S2B and S2C), we conclude that simultaneous engagement of the I44 patches of both Ub^prox^ and Ub^dist^ is essential to properly position the scissile bond for MINDY1 and MINDY2 to cleave K48 chains. Comparison of all of the available structures of DUBs in complex with diUb chains reveals that this mode of symmetric binding, where the I44 patches of both Ub^prox^ and Ub^dist^ are engaged with the DUB, is unique to MINDY1/2 (Figure 1A, inset; Table S1). K48-linked chains exist in a dynamic equilibrium between closed and open conformations (Ye et al., 2012); however, the extended conformation adopted by K48-linked diUb, when bound to the catalytic domains of MINDY1 and MINDY2, is distinct from all previously reported conformations of K48-linked chains (Figures S2G and S2H). To our knowledge, these structures of MINDY1/2 represent the first crystal structures of any DUB in complex with K48-linked Ub chains.

### Cys loop regulates DUB activity

**Figure 2:**
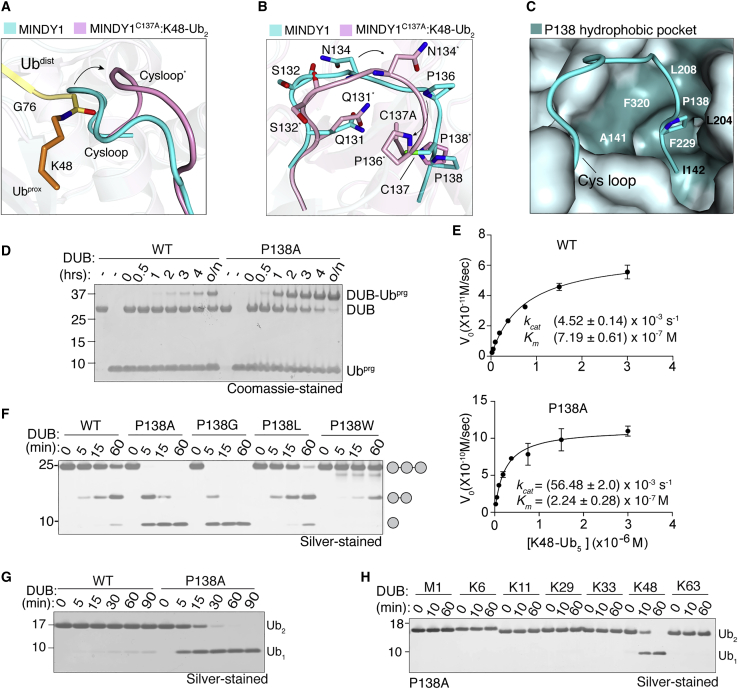
Cys loop mobility regulates DUB activity (A) Representation of the Cys loop in a superposition of MINDY1^apo^ (cyan) and MINDY1^C137A^:K48-Ub_2_ complex (pink). The isopeptide bond between K48 of Ub^prox^ (orange) and G76 of Ub^dist^ (yellow) is shown in sticks. (B) Close-up view of (A) showing amino acid side chain rearrangements (side view). (C) Surface representation of the hydrophobic pocket in MINDY1^apo^ that accommodates the Cys loop residue P138. (D) Coomassie-stained gel comparing activity of MINDY1 WT and P138A to Ub^Prg^ in a time course. (E) Steady-state kinetics of K48-linked pentaUb cleavage by MINDY1 WT and P138A mutant derived from reactions with varying concentrations of fluorescently labeled Ub_5_ (n = 2; means ± SDs). (F) Silver-stained gel comparing cleavage of K48-Ub_3_ by MINDY1 WT and indicated mutants. (G) DUB assay comparing cleavage of K48-Ub_2_ by MINDY1 WT and P138A mutants. (H) DUB assay monitoring cleavage of diUb of 7 different linkage types by MINDY1 P138A. See also Figure S3.

In MINDY1 and MINDY2, a flexible loop (T130 to P138 in MINDY1 and T259 to P267 in MINDY2) connects β2 to α1, which we term the Cys loop since the catalytic cysteine sits at its base. In the apo-form of the DUB, this Cys loop occludes the catalytic center and would sterically hinder positioning of the scissile bond across the active site (Figure 2A). Comparing the structures of the MINDY1^C137A^:K48-Ub_2_ complex with MINDY1^apo^ does not show any large-scale conformational changes induced within the catalytic domain upon Ub binding (RMSD 1 Å over 244 aligned Cα atoms) (Figure S3A). The only significant change is in the Cys loop that undergoes significant remodeling, during which several hydrogen bonds are broken, accompanied by the formation of new bonds (Figures 2B and S3B–S3D). This extensive interaction network and remodeling of the Cys loop is also observed in MINDY2 (Figures S3E and S3F), and when restructured, the Cys loop no longer impedes Ub binding. In the apo structure, in addition to the obstructing Cys loop, the catalytic residues are misaligned in an unproductive conformation (Abdul Rehman et al., 2016). Hence, the apo state of the enzyme corresponds to an inactive, autoinhibited conformation and the DUB transitions to an active state, when in complex with K48-linked diUb.

The catalytic cysteine in both MINDY1 and MINDY2 are flanked by proline residues: MINDY1 (P136-C137-P138) and MINDY2 (P265-C266-P267). When we compare Cys loop conformations in the autoinhibited and active states of MINDY1, P138 in the Cys loop remains fixed, while the rest of the loop moves (Figure 2B). P138 sits in a hydrophobic pocket formed by A141, I142, L204, L208, F229, and F320 to function as an anchor point for the Cys loop (Figures 2C and S3C). When in complex with diUb, the nature of the interactions of P138 within the hydrophobic pocket change, with only L204, L208, and F320 mediating interactions, whereas F229 and I142 now no longer interact with P138 (Figure S3D). In contrast to the anchored P138, the other flanking proline, P136, shows significant movement upon complex formation (Figure 2B). P136 does not interact with neighboring residues in the apo state; however, in the diUb-bound active state, P136 is part of a strong intramolecular interaction network consisting of hydrophobic interactions with Y114, L139, L140, and A205 (Figures S3C and S3D).

Based on these observations, we predict that P138 modulates the dynamics of the Cys loop, as it provides rigidity to the loop by anchoring itself into the hydrophobic pocket. Hence, the mutation of P138 to alanine should dislodge the hydrophobic pocket interactions and result in a more dynamic, flexible loop. To test this possibility, we determined the crystal structures of the MINDY1 P138A mutant on its own and in complex with K48-Ub2. As hypothesized, the absence of P138 results in a less-extensive engagement with the hydrophobic pocket, resulting in a more mobile Cys loop, as suggested by higher b-factors of its residues (Figures S3G–S3K). To determine the effect of a more flexible Cys loop on DUB activity, we assayed the reactivity of MINDY1 toward Ub^Prg^. In contrast to the WT enzyme, the P138A mutant was readily modified by Ub^Prg^, thereby supporting our notion that the P138A mutation makes the Cys loop more flexible to reduce the steric hinderance to Ub binding (Figure 2D). Next, we determined enzyme kinetics using fluorescently labeled K48-linked pentaUb, which revealed that MINDY1 P138A mutant is a much more active enzyme with >10-fold higher *k*_*cat*_ and ∼3-fold lower *K*_*m*_ compared to MINDY1 WT (Figure 2E). As the Cys loop sterically interferes with polyUb binding, an increase in loop flexibility may account for the observed reduction in *K*_*m*_.

To test the role of the hydrophobic pocket in keeping P138 anchored, we mutated P138 to smaller residues (A or G), which would disrupt anchoring, or to bulky hydrophobic residues (L or W), which would lock the Cys loop in the inhibited state. A chain cleavage assay with these mutants shows that disrupted anchoring (P138A or P138G) increases chain cleavage, whereas increased hydrophobicity of the side chain impairs DUB activity (Figure 2F). The consequence of a more flexible loop is also underscored by the ability of MINDY1 P138A to cleave diUb, which MINDY1 WT is unable to cleave (Figure 2G). Similarly, mutating the equivalent residue in MINDY2 (P267A) also leads to increased DUB activity (Figures S3L and S3M). Fittingly, Miy2 (Ypl191C), the yeast ortholog of MINDY2, has an alanine at this position (A29), which may explain why Miy2 is a much more active enzyme compared to MINDY1 (Abdul Rehman et al., 2016). Importantly, increasing Cys loop flexibility and thereby catalytic activity with the P138A mutation does not change the linkage specificity of MINDY1 for K48 chains, as the mutant only cleaves K48 chains and no other linkage type (Figure 2H). Collectively, these results demonstrate that the Cys loop regulates MINDY1 catalytic activity, but not linkage selectivity.

### Mechanism of autoinhibition

**Figure 3:**
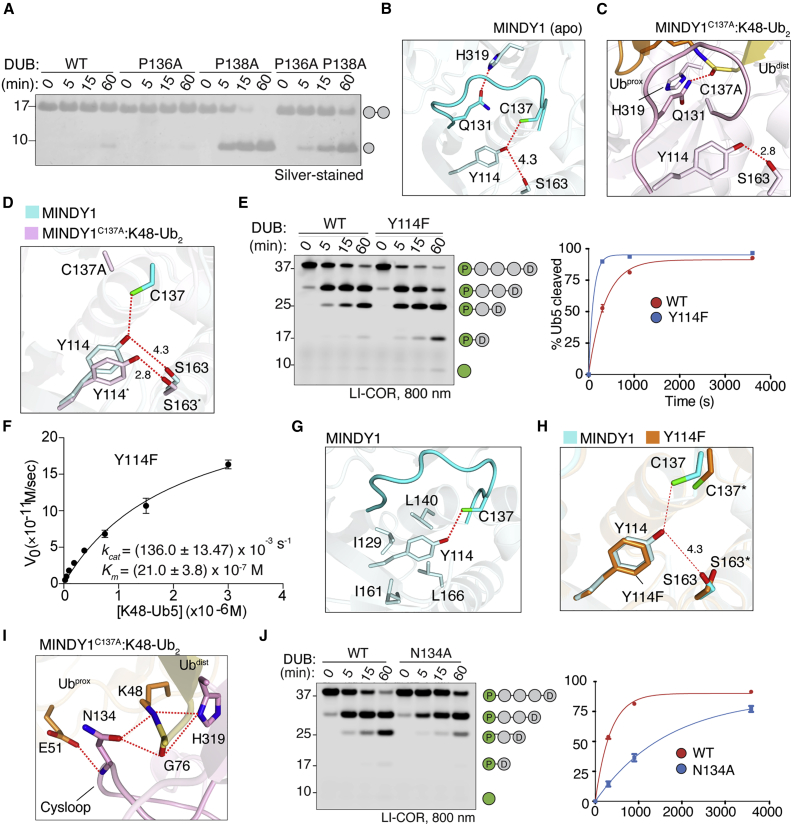
Autoinhibition and activation of MINDY1 (A) DUB assay monitoring the cleavage of K48-Ub_2_ by MINDY1 and indicated mutants. (B) Close-up view of catalytic residues and their interactions in MINDY1 (apo). C137 is out of plane with H139 and is hydrogen bonded with Y114 in MINDY1 (apo). Red dotted lines indicate hydrogen bonds. (C) Close-up view as in (B) for the MINDY1^C137A^:K48-Ub_2_ complex. The catalytically productive state conformation leads to the formation of new sets of bonds as shown. The oxyanion hole residue Q131, which was in contact with catalytic H319 in (B), now forms interactions with the carbonyl of the incoming scissile bond. (D) Lateral movement of Y114 and its interactions in MINDY1 (apo) and MINDY1^C137A^:K48-Ub_2_ complex. (E) DUB assays comparing cleavage of fluorescently labeled pentaUb by MINDY1 and Y114F mutant. The percentage hydrolysis of pentaUb is plotted against time (right). (F) Steady-state kinetics of K48-linked pentaUb cleavage by MINDY1 Y114F (n = 2; means ± SDs). (G) Close-up view of Y114 (phenyl ring) interactions with hydrophobic residues on adjoining secondary structure elements in MINDY1 (apo). (H) A close-up image of active site of apo MINDY1 Y114F mutant compared to WT. Hydrogen bonding of C137 to Y114 is broken in the mutant. (I) Interactions of Cys loop residue N134 in stabilizing the isopeptide bond for catalysis. (J) Cleavage of pentaUb chains fluorescently labeled on Ub^prox^ by MINDY1 WT and N134A mutant. The panel on the right shows the quantification of the DUB assay. n = 2; mean ± SD. See also Figure S4.

Mutation of the other Cys-flanking proline P136, MINDY1 P136A, is unable to cleave diUb, whereas the P138A mutant is very active (Figures 2G and 3A). However, the double-mutant MINDY1 P136A/P138A shows diminished activity compared to the P138A mutant, suggesting a regulatory role for P136. One notable intramolecular interaction of P136 is with the phenyl ring of Y114 (Figure S3D). In the autoinhibited state of MINDY1, sulfur-centered hydrogen bonding of the catalytic C137 with the hydroxyl of Y114 rotates C137 away from the active site (Figure 3B). In the active conformation, this inhibitory Y114-C137 interaction is broken due to the Cys loop movement, and the side chain of Y114 now hydrogen bonds with S163 (Figures 3C and 3D). Interestingly, in the MINDY1∼Ub complex, which represents the product-bound intermediate state, Y114 is hydrogen bonded to S163 before the transition back from the activated to the inhibited state after Ub chain hydrolysis (Figure S4A). These observations strongly imply a linchpin role for Y114 in regulating the transition from an inhibited to active state. Disruption of this single hydrogen bond with a Y114F mutation results in increased DUB activity, with an ∼30-fold increase in *k*_*cat*_ compared to WT (Figures 2E and 3E–3H). A conserved mechanism operates in MINDY2, as mutation of the analogous residue Y243 also leads to increased DUB activity (Figures S4B and S4C). Y114 is surrounded by hydrophobic residues, which interact and stabilize the position of the phenyl ring, and in fact, the MINDY1 Y114A mutant is less active compared to WT and the Y114F mutant (Figures 3G, 3H, and S4D).

In addition to P138 and Y114, the inhibitory conformation of the Cys loop is further maintained by the intramolecular interaction between N134 and S132 in MINDY1 (Figure S3B). When in complex with K48-Ub_2_, this interaction is broken as N134 now interacts with K48, G76, and E51 of Ub^prox^ (Figure 3I). These interactions of the side chain of N134 with the substrate are important for deubiquitylation activity, as mutating N134 to alanine impairs the catalytic activity of MINDY1 (Figure 3J). This suggests a model of substrate-assisted catalysis, where interaction of S132 and N134 of MINDY1 with residues in Ub enable the transition of MINDY1 from an inhibited to an active enzyme.

In the autoinhibited state of MINDY1, the oxyanion hole forming Q131 is hydrogen bonded to the catalytic H319 (Figure S4E). In the active state observed in the MINDY1^C137A^: K48-Ub_2_ complex, N134 and the catalytic H319 both form hydrogen bonds with K48 (Figure 3I) and Q131 interacts with the isopeptide bond (Figure S4F). In addition to the interactions with Ub^prox^ that stabilize the catalytic site in a productive conformation, Ub^prox^ also stabilizes the binding of Ub^dist^ onto the S1 site (Figure S2A). Hence, in a substrate-activated mechanism, the binding of K48-linked polyUb stabilizes the productive conformation of the catalytic site in MINDY1 and MINDY2. In the absence of Ub^prox^ in the MINDY1∼Ub structure, both H319 and Q131 exist in two alternate conformations. In one conformation, H319 is hydrogen bonded to Q131, similar to the inhibited state; however, in a second conformation, Q131 is flipped out and does not interact with any neighboring residue (Figure S4G). In contrast, in MINDY2^apo^, the catalytic H448 is flipped out, and Q260 is not able to form a hydrogen bond (Figures S4H and S4I). However, the binding of Ub^prox^ brings H448 closer to the catalytic C266 to form a productive active site (Figure S4J). In summary, the Cys loop dynamics and the interactions with K48-linked chains modulate the transition of MINDY1 and MINDY2 from an inhibited to an active state.

### Non-canonical catalytic mechanism

**Figure 4:**
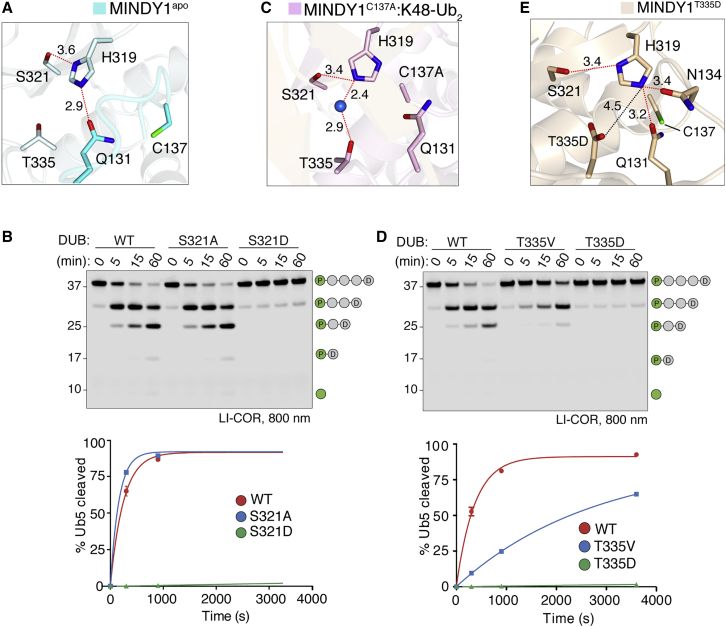
MINDY1 uses a non-canonical catalytic mechanism (A) Close-up view of the catalytic site in MINDY1 apo. The dotted red lines indicate hydrogen bonds, the dotted black line the ionic bond, and the blue sphere indicates water molecule. (B) DUB assay comparing the cleavage of fluorescently labeled K48-Ub_5_ by MINDY1 WT, S321A, and S321D mutants. The percentage of pentaUb hydrolyzed is plotted against time (right). n = 2; mean ± SD. (C) Close-up view of the catalytic site in MINDY1^C137A^:K48-Ub_2_ complex. (D) DUB assay as in (B) comparing the chain cleavage by MINDY1 WT, T335V, and T335D mutants. The percentage of pentaUb hydrolyzed is plotted against time (right). n = 2; mean ± SD. (E) Close-up view of the catalytic site in MINDY1^T335D^. See also Figure S5.

In most thiol proteases, a third catalytic residue, usually an Asp or Asn, serves to correctly position the catalytic His (Clague et al., 2019). In all of the determined structures of MINDY1 and MINDY2, the identity of this third catalytic residue is unclear. In the MINDY1^C137A^:K48-Ub_2_ structure, S321 is 3.6 Å away from H319 and could function as a catalytic residue, as described for USP30 (Figure 4A; Gersch et al., 2017; Sato et al., 2017). However, to our surprise, MINDY1 S321A mutation did not abolish activity, but instead resulted in a modest increase in MINDY1 activity (Figure 4B). A search for other potential residues only revealed a distant T335 situated ∼6 Å away (Figure 4A). Despite this distance, we found a water molecule to coordinate T335 with H319 via hydrogen bonding, an unusual mechanism that may serve to orient H319 for catalysis, thereby adopting the function of the third catalytic residue (Figures 4C and S5A). To test whether such an unusual catalytic architecture is possible, we introduced a T335V mutation to disrupt this water bridge, which resulted in a substantial loss of activity (Figure 4D). We conclude that T335 competes with S321 to correctly position H319 for catalysis, thus adding another layer of regulation. In this scenario, S321 functions as an inhibitory residue, explaining why the S321A mutation enhances activity. Further supporting this model is the mutation of S321 to aspartate, which completely abolishes the catalytic activity, as a strong ionic bond between S321D and the catalytic histidine (H319) likely blocks the catalytic function of H319, thus rendering the DUB inactive (Figure 4B).

As T335 serves a catalytic function to correctly position H319 for catalysis, we wondered if mutating T335 to a negatively charged residue would enhance the activity of MINDY1. To our surprise, the mutation of T335 to aspartate also abolishes MINDY1 activity (Figure 4D). To understand the reason underlying this loss of activity, we determined the crystal structure of MINDY1^T335D^, which revealed the formation of an ionic interaction between T335D and the catalytic H319. H319 is locked in this conformation, which is further rigidified by the network of hydrogen bonds with Q131, S321, and N134, resulting in the catalytic Cys being rotated away from H319 (Figures 4E and S5B). Hence, the mutation T335D also forces the enzyme into an unproductive catalytic state, thus highlighting the requirement of a water molecule to bridge an interaction with T335 via hydrogen bonding to correctly position H319. MINDY2 also uses a similar non-canonical catalytic architecture in which T464 is the third catalytic residue and S450 serves as a competitive element (Figures S5C–S5E).

### Why does MINDY1 preferentially cleave long Ub chains?

The minimal catalytic domains of both MINDY1 and MINDY2 show higher activity at cleaving longer chains as revealed by DUB assays with fluorescently labeled polyUb chains of increasing length (Figures S6A and S6B). We hypothesize that the preference of these DUBs for cleaving longer chains is due to the presence of additional Ub binding sites within the catalytic domain. If MINDY1 and MINDY2 do have additional Ub binding sites, we predict that they would have higher affinity for longer K48 chains compared to K48-Ub_2_. Hence, we performed isothermal calorimetry (ITC) measurements in which catalytically dead MINDY1 C137A was titrated into K48-diUb, triUb, tetraUb, or pentaUb (Figure S6C). While there was no measurable binding for diUb by ITC, we observed an increase in binding affinity with increasing chain length. MINDY1 binds to triUb with an affinity of ∼6 ± 3 μM, which increases to ∼1 ± 0.2 μM for tetraUb and to ∼250 nM for pentaUb. To determine whether MINDY1 is specific not only at cleaving K48-linked chains but also at recognizing them, we tested whether K29- and K33-tetraUb, chains not cleaved by MINDY1, could bind to it. These ITC measurements revealed that MINDY1 C137A does not bind K29- and K33-linked chains (Figure S6D). These results indicate that the arrangement of the Ub binding sites on the enzyme permit only K48-linked chains to bind, and MINDY1/2 has at least five distinct Ub binding sites within its catalytic domain.

**Figure 5:**
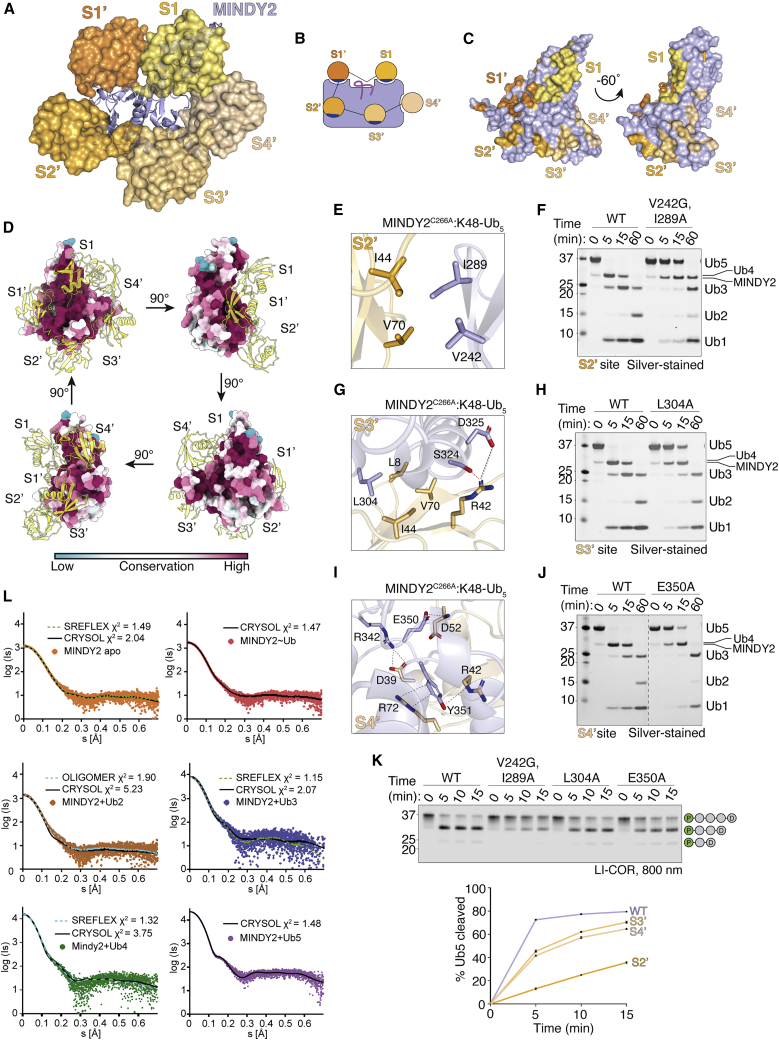
Crystal structure of MINDY2 in complex with K48-linked pentaUb (A) The MINDY2^C266A^:K48-Ub5 complex crystal structure with MINDY2 (light blue). Ub molecules are depicted with transparent surfaces (S1: yellow orange, S1′: tv-orange, S2′: bright orange, S3′ light orange, S4′: wheat). (B) Schematic representation of MINDY2^C266A^:K48-Ub5 complex with Ile44 patches on Ub involved in binding shown in blue. (C) Surface representation of MINDY2, with the footprint of each Ub highlighted at each Ub-binding site. (D) Surface conservation analysis of MINDY2 from metazoan ortholog sequences. MINDY2 is shown as a surface model, rotated by 90° in each view, and the surface residues are colored by conservation score. The pentaUb chain is shown as a yellow ribbon model, and the 5 Ub binding sites on MINDY2 are annotated. (E) Close-up of key residues at the S2′ site and their interactions with Ub. (F) DUB assay comparing cleavage of K48-Ub5 by MINDY2 WT and the indicated S2′ site mutants. (G) Close-up of key residues at the S3′ site and their interactions with Ub. (H) DUB assay comparing cleavage of K48-Ub5 by MINDY2 WT and indicated S3′ site mutants. (I) Close-up of key residues at the S4′ site and their interactions with Ub. (J) DUB assay comparing cleavage of K48-Ub5 by MINDY2 WT and indicated S4′ site mutants. Dashed line: gel truncation to exclude mutants irrelevant to this study. (K) DUB assay comparing cleavage of fluorescently labeled K48-Ub5 by MINDY2 WT and S2′, S3′, and S4′ mutants. n = 3; mean ± SEM. (L) SAXS curves of MINDY2, apo molecule, and in complex with monoUb, K48-linked Ub_2_, Ub_3_, Ub_4_, and Ub_5_, respectively, and their fits computed from atomic models by CRYSOL. For Mindy2-Ub_3_, normal mode analysis (NMA) with SREFLEX was used for the refinement of the expected atomic models. For Mindy2-Ub_2_, OLIGOMER was applied on atomic models in which Ub_2_ occupying positions S1 and S1′ or S1 and S3′ were used to quantify their mixture. See also Figures S6 and S7.

To conclusively establish whether the catalytic domain contains five Ub binding sites, we crystallized the catalytic domain of MINDY2 in complex with K48-Ub_5_. The structure of the complex reveals 5 Ub molecules wrapped around the catalytic domain of MINDY2, resembling a pearl necklace (Figures 5A and 5B; Table S4). All Ub binding sites show high conservation among metazoa, with the S1 and S1′ sites being particularly highly conserved (Figures 5D and S6E). A closer look at how Ubs bind to the S2′-S3′ sites reveals that they are primarily centered around the I44 patch of Ub. Interaction with Ub at the S2′ site is mediated via V242 and I289 of MINDY2 (Figure 5E). At the S3′ site, L304 engages with the Il44 patch of Ub and both S324 and D325 interact with R42 via hydrogen bonding and ionic bonding, respectively (Figure 5G). In contrast to the other sites, Ub binding at the S4′ site does not involve the I44 patch and is instead primarily mediated by hydrogen bonding between E350 of MINDY2 and the backbone of D52 of Ub, ionic interactions of R342 with D52 of Ub and cation-π stacking between Y351 and R72 of Ub (Figure 5I). Mutating key residues at each site on MINDY2 to weaken the interaction with Ub impedes the ability of MINDY2 to cleave pentaUb (Figures 5F, 5H, 5J, and 5K). Furthermore, mutating residues at equivalent positions in MINDY1 also inhibit cleavage of Ub5 (Figures S6F and S6G). These experiments highlight the importance of Ub interactions at all five Ub binding sites on MINDY1^cat^ and MINDY2^cat^ for efficient cleavage of K48-polyUb.

To validate the presence of 5 distinct Ub binding sites on MINDY2, we performed small-angle X-ray scattering (SAXS) analyses on different MINDY2 complexes. SAXS data were collected for MINDY2 on its own, covalently linked to Ub^Prg^ and in complex with K48-Ub_2_, Ub_3_, Ub_4_, and Ub_5_ (Figure S7A; Table S2). The SAXS curves were first computed from all of the working models by using CRYSOL (Svergun et al., 1995) and compared with the experimental data. For both MINDY2 apo and MINDY2∼Ub complex, computed data from the models yielded good agreement with the experiment, confirming the two structures in solution (Figures 5L and S7A; Table S2). For complexes with Ub chains, some deviations were observed (Figures 5L and S7B–S7E), and the expected models were further refined to fit the SAXS data. The SAXS data of the MINDY2^C266A^:K48-Ub_2_ complex were analyzed for the presence of complexes with distinguishable Ub_2_ binding positions with OLIGOMER (Konarev et al., 2003). The best fit was obtained for a mixture of two different binding modes: productive (∼15%), where Ub moieties are bound in the S1 and S1′ sites, and unproductive (∼85%), where they are bound in the S1 and S3′ sites (Figures 5L and S7B). Interestingly, this unproductive mode resembles the K48-Ub_2_ conformation recognized by OTUB1 (Juang et al., 2012; Wiener et al., 2012; Figure S2H). The higher prevalence of Ub binding in the unproductive mode possibly explains the inefficient cleavage of K48-Ub_2_ by MINDY1 and MINDY2. Importantly, the SAXS data for the MINDY2^C266A^:K48-Ub_5_ complex are in good agreement with the obtained crystal structure, with a χ^2^ value of 1.48 (Figure 5L), validating that the conformations observed in the crystal structure and in solution are similar (Figure S7E). In summary, the enzyme assays, ITC measurements, SAXS analyses, and crystal structures convincingly show that five distinct Ub binding sites are present within the minimal catalytic domain.

### Ubiquitin chain length determines exo- or endo-cleavage

We had previously demonstrated that MINDY1 is an exo-DUB that cleaves pentaUb from the distal end of the chain to release one Ub moiety at a time from the end of the chain (Abdul Rehman et al., 2016). One mechanism to achieve such directionality of cleavage would be that MINDY1 recognizes the distal end of the chain. However, close examination of the MINDY2^C266A^:K48-Ub_5_ structure revealed that the K48 residue of Ub^dist^ is solvent exposed, which implies that this Ub does not necessarily have to be the extreme distal moiety in a chain (Figure S8A). MINDY1/2 could therefore, in principle, possess endo-activity and cleave within a Ub chain. As we here identified five Ub binding sites within the catalytic domain, we carefully analyzed how MINDY1/2 would cleave chains containing more than five Ub moieties.

**Figure 6:**
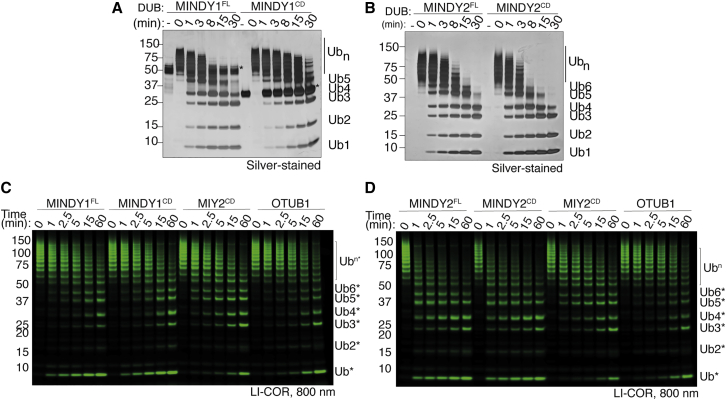
Ubiquitin chain length determines exo- and endo-cleavage activities (A) Silver-stained gels of DUB assays monitoring cleavage of long K48-linked polyUb chains containing >6 Ub moieties by MINDY1^FL^ and MINDY1^cat^. (B) As in (A), but comparing MINDY2^FL^ and MINDY2^cat^. (C) DUB assay monitoring cleavage of distally labeled longer polyUb chains by MINDY1^FL^ and MINDY1^cat^ with 2 known endo-DUB controls: MIY2 and OTUB1. (D) As in (D), but comparing MINDY2^FL^ and MINDY2^cat^.

When long K48-linked chains containing more than five Ub moieties were incubated with MINDY1/2, these long chains collapsed to predominantly form a mixture of chains containing up to four Ub molecules (Figures 6A and 6B). Compared to the long chains of five or more Ubs, tetraUb is not a preferred substrate and rapidly accumulates initially, which is further processed by gradual cleavage only at later time points. To confirm our observation that MINDY1/2 may not be exo-DUBs when presented with long Ub chains, we generated longer K48-linked polyUb chains (>7 Ub), each labeled with a fluorophore only at the extreme distal Ub moiety. If MINDY is a strict exo-DUB, it would cleave these chains from the distal end to yield only fluorescent monoUb as the main product at early time points, and no other fluorescent species (Ub2–Ub6) should be formed. However, we observe that both MINDY1 and MINDY2 rapidly produce a range of fluorescent cleavage products that lie between Ub1 and Ub7, suggesting endo-activity as the DUB can cleave anywhere within the polyUb chain, resulting in the formation of chains of all intermediate lengths as products (Figures 6C and 6D). These results suggest that MINDY1/2 can work as endo-DUBs to cleave within long chains (>5 Ub), but act as exo-DUBs on shorter chains (<6 Ub) to cleave the distal Ub. Hence, in addition to being specific for K48-linked polyUb, MINDY1 and MINDY2 also sense Ub chain length to position and cleave long K48-linked chains down to tetraUb.

## Discussion

**Figure 7:**
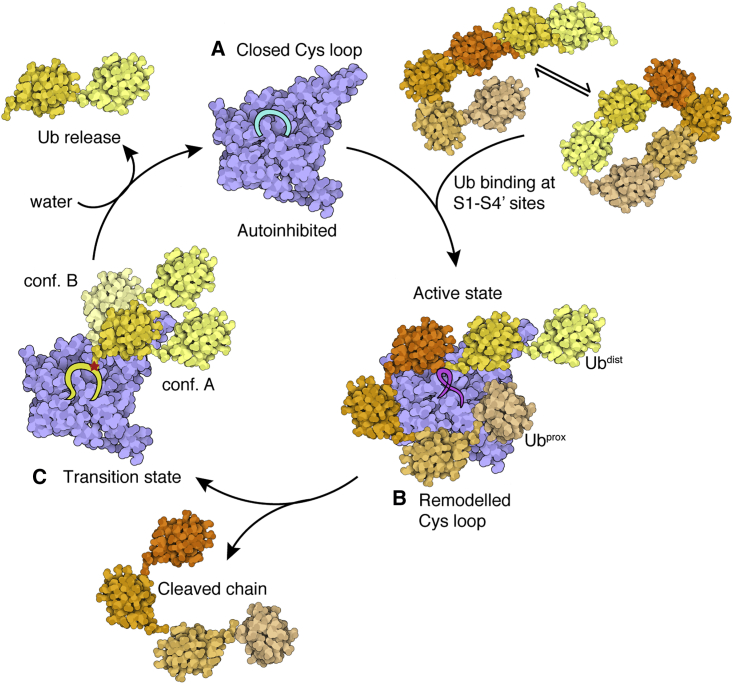
Model summarizing the catalytic mechanism of K48-linked polyUb (A) MINDY1 and MINDY2 exist in an autoinhibited conformation in which the Cys loop is in a closed conformation that sterically interferes with Ub binding and also contributes to keeping the catalytic site inhibited. (B) In a substrate-driven mechanism, Ub interactions release inhibition and activate the DUB, resulting in chain cleavage and release of the Ub chain. (C) In the product intermediate transitional tetrahedral state, the Ub occupies the S1 site. As this is not a strong binding interface, this Ub exists in 2 different conformers. Attack by a water molecule releases the Ub and returns the DUB to an inhibited conformation. Created using Illustrate (Goodsell et al., 2019). See also Figure S8.

The three crystal structures of MINDY1 reveal distinct states in the catalytic cycle of MINDY1, namely, autoinhibited (Apo), substrate-bound active state (MINDY1^C137A^:K48-Ub_2_) and the product-bound intermediate state (MINDY1∼Ub) (Figures 7 and S8B). In the apo state, the Cys loop mediates autoinhibition and sterically interferes with Ub binding. While large-scale conformational changes are not observed upon enzyme:substrate complex formation, Ub binding at the S1′ site releases the autoinhibition mediated by the Cys loop to stabilize Ub binding at the S1 site. Hence, in a model of substrate-driven activation, Ub bound at the S1′ site interacts with the Cys loop to drive the transition of MINDY1 and MINDY2 from inhibited to active enzymes.

In MINDY1 and MINDY2, the correct positioning of the scissile bond requires Ub binding at both the S1 and S1′ sites. In several DUBs, polyUb recognition and cleavage depends on extensive Ub interactions at the S1 site (Leznicki and Kulathu, 2017). When binding at the S1 site is not strong, DUBs rely on additional interactions commonly at the S1′ site or S2 sites (Békés et al., 2015; Flierman et al., 2016; Ye et al., 2011). This is a hallmark of DUBs such as CYLD and OTU family DUBs, where Ub interactions at the S1′ site place the proximal Ub in a position that orients a specific lysine into the catalytic site, thereby conferring linkage specificity (Licchesi et al., 2011; Mevissen et al., 2016; Sato et al., 2015). A similar mechanism drives linkage selectivity in MINDY1 and MINDY2, where the proximal Ub is positioned in such a way that only K48-linked polyUb can access the catalytic groove. Our SAXS data show that a significant proportion of diUb binds the DUB in a non-productive conformation at the S1 and S3′ sites, which suggests another regulatory layer to select for long polyUb chains that can simultaneously engage with all Ub binding sites on the DUB and properly position the scissile bond for cleavage. MINDY1 has a weak affinity for K48-linked diUb and the steric hindrance imposed by the Cys loop prevents diUb binding and therefore cleavage. The P138A mutation, which increases the mobility of the Cys loop not only enables transition to the active conformation but also facilitates Ub binding as evidenced by lower *K*_*m*_ for pentaUb and binding to diUb.

PolyUb chains, including K48-linked chains, have been observed in different conformations and are dynamic in solution, existing in an ever-changing equilibrium between closed and open conformations (Cook et al., 1992; Eddins et al., 2007; Hirano et al., 2011; Lai et al., 2012; Lu et al., 2020; Trempe et al., 2010; Ye et al., 2012). Our structures reveal that the K48 chains adopt an extended conformation when positioned across the active site of MINDY1/2. Furthermore, we observed pentaUb wrapping around MINDY2 like a pearl necklace, in an open conformation where the I44 patches of all Ub moieties, except the most proximal Ubs, engage with the DUB for binding. Of note, our data suggest that K48-linkage specificity is also maintained at the proximal end, as other chain types tested do not bind to MINDY1. Despite the range of conformations that can be adopted by Ub chains, it is remarkable how enzymes and binding domains have evolved mechanisms to selectively recognize polyUb of a particular linkage type and length.

The most favorable polyUb binding mode that results in the most efficient cleavage is that all Ub-binding sites on the DUB are occupied. Therefore, MINDY1/2 can efficiently bind a K48-Ub chain of length n (with n ≥ 5) in n-4 preferred ways within the chain, resulting in a rapid collapse of longer chains to tetraUb. Starting with pentaUb, the minimum chain length that can satisfy the binding requirements, an exo-form of cleavage, is forced, with one Ub monomer being trimmed from the chain with each cleavage event. As the chains become shorter (<5 Ub), all five binding sites can no longer be occupied, thus leading to a decrease in cleavage efficiency. TetraUb, for instance, does not occupy all of the binding sites on the DUB, and consequently cleavage is inefficient, resulting in the observed accumulation of Ub_4_ (Figures 6A–6D). We therefore propose that MINDY1 and MINDY2 primarily act as endo-DUBs to rapidly recognize and trim long Ub chains down to tetraUb. An emerging theme in ubiquitylation is a role for the length of the polyUb chain in determining the consequence of the post-translational modification. For instance, the protease DDI2 will cleave the transcription factor NRF1 only when it is modified with long polyUb chains (Dirac-Svejstrup et al., 2020). Similarly, the unfoldase Cdc48/p97-Ufd1-Npl4 has several Ub binding sites and efficiently unfolds Mcm7 only when it is modified with K48 chains containing at least five Ubs (Deegan et al., 2020; Twomey et al., 2019).

Our studies of MINDY1 and MINDY2 reveal two remarkably similar enzymes, with analogous regulatory mechanisms and substrate specificities. However, MINDY1 and MINDY2 are modular DUBs that, in addition to their catalytic domain, possess N-terminal regions of unknown function and distinct C-terminal tandem MIU domains (tMIU) (Abdul Rehman et al., 2016), and potential differences in the biological roles of MINDY1 and −2 are likely to arise from these additional domains. The tMIU of MINDY1 is highly selective at binding to K48-linked polyUb chains, with MIU2 of the tandem motif being the main determinant of this specificity (Kristariyanto et al., 2017). In contrast, the tMIU of MINDY2 is non-specific and binds to polyUb chains of different linkage types. This raises the possibility that MINDY2 cleaves polyUb containing mixed or branched Ub linkages, where the tMIU binds to the non-K48-linked part of the chain and the DUB cleaves the K48 linkages, suggestive of a cellular function distinct from MINDY1.

Crystal structures of many DUBs reveal that their catalytic residues are often in unproductive conformations in the absence of substrate, and conformational rearrangements are triggered by Ub binding, leading to realignment of the catalytic residues into a productive conformation (Boudreaux et al., 2010; Hu et al., 2002; Keusekotten et al., 2013; Mevissen et al., 2016; Sato et al., 2015). Similarly, we observed Ub binding to MINDY1/2 to induce several structural rearrangements leading to a functional active site. Most thiol DUBs feature the canonical catalytic triad composed of Cys, His, and Asp/Asn (Ronau et al., 2016). The Asp/Asn residue plays a secondary role by properly orienting the His in the catalytic triad. DUBs such as USP16, USP30, and USP45 have a serine in place of the Asp/Asn, making them distinct from other DUBs (Gersch et al., 2017; Sato et al., 2017). MINDY1 features an atypical catalytic triad in which a Thr residue orients the His via a water bridge. Intriguingly, a local non-catalytic Ser residue plays an inhibitory role by competing with the catalytic Thr for interaction with the catalytic His and improperly orienting it. This is reminiscent of OTULIN, in which inhibitory interactions mediated by an Asp with the catalytic His inhibit the DUB and are relieved upon substrate binding (Keusekotten et al., 2013). In MINDY1 and MINDY2, an additional layer of regulation is imparted by a sulfur-centered hydrogen bond between a Tyr and the catalytic Cys, which further reinforces autoinhibition.

In summary, our work reveals that MINDY1 and MINDY2 are specialized DUBs that sense both Ub chain length and linkage type. The remarkable specificity that MINDY1 and MINDY2 possess at cleaving K48-linked chains to trim long polyUb chains may help reveal the cellular functions of these evolutionarily conserved DUBs. That MINDY1 and MINDY2 have evolved so many layers of regulation and activation steps suggests key regulatory functions for these DUBs.

### Limitations of the study

Our work reveals the mechanisms of autoinhibition and activation in MINDY1 and MINDY2 and the recognition of long Ub chains as preferred substrates. It is intriguing that MINDY1 and MINDY2 possess such remarkable chain-trimming activity; however, without insights into the identity of the substrates of these enzymes, it is difficult to establish the significance of the activity of MINDY1/2 in pruning polyUb down to tetraUb. While we show the presence of five distinct Ub binding sites on the catalytic domain of MINDY, our analyses do not reveal whether there is a specific order in which the Ub moieties within the chain bind. Despite these limitations, our work reveals fundamental insights into the mechanism of MINDY1 and MINDY2. Furthermore, our characterization will allow these enzymes to be used as valuable tools to study Ub signaling, especially to probe the role of Ub chain length in eliciting cellular responses.

## STAR★Methods

### Key resources table

**Table:** 

REAGENT or RESOURCE	SOURCE	IDENTIFIER
**Bacterial strains**		

*E.coli* BL21-CodonPlus (DE3)-RIL	Agilent	Cat# 230245

**Chemicals, peptides, and recombinant proteins**		

Propargylamine	Sigma Aldrich	Cat# P50900-5G
Ampicillin sodium	Formedium	Cat# AMP100
Gluthahione Sepharose 4B	Abcam	Cat# ab193267
Protease inhibitor cocktail tablets	Thermo Scientific	Cat# A32965
AEBSF	Melford Laboratories Ltd	Cat# A20010
IRDye 800CW Maleimide	Li-Cor	Cat# 929-80020
Precision Plus Protein™ standards	Bio-Rad	Cat:# 1610373
Pierce™ silver stain kit	Thermo Scientific	Cat# 24612
InstantBlue protein stain	Abcam	Cat# 119211
Ubiquitin	This study	N/A
MINDY1 and mutants	This study	N/A
MINDY2 and mutants	This study	N/A
E1, E2 enzymes	This study	N/A

**Recombinant DNA**		

	This study	Table S3 (https://mrcppureagents.dundee.ac.uk/)

**Deposited data**		

Structure factor and coordinates files	RCSB-PDB	MINDY1^C137A^:K48-Ub_2_: 6TUV, MINDY2^C266A^:K48-Ub_2_: 6Z7V, MINDY1^P138A C137A^:K48-Ub_2_: 6TXB, MINDY1^Y114F^: 6YJG, MINDY1^T335D^: 6Y6R, MINDY1^P138A^: 6Z90, MINDY2^apo^: 6Z49, MINDY2^C266A^:K48-Ub_5_: 7NPI.
SAXS data	SASBDB	MINDY2^apo^: SASDJ93, MINDY2-Ub^Prg^: SASDJA3, MINDY2^C266A^:K48-Ub2: SASDJB3, MINDY2^C266A^:K48-Ub3: SASDJC3, MINDY2^C266A^:K48-Ub4: SASDJD3, MINDY2^C266A^:K48-Ub5: SASDJE3.
Original gel Scans	Mendeley Data	https://doi.org/10.17632/f6zfttw8zg

**Software and algorithms**		

Prism	Graphpad	https://www.graphpad.com/scientific-software/prism/
Image Studio Lite	LI-COR	https://www.licor.com/bio/image-studio-lite/
XDS	Kabsch, 2010	http://xds.mpimf-heidelberg.mpg.de/https://xds.mr.mpg.de
AIMLESS	Evans and Murshudov, 2013	https://www.ccp4.ac.uk/html/aimless.html
CCP4 interface version 7.0.04	Winn et al., 2011	https://www.ccp4.ac.uk/
Phenix	Liebschner et al., 2019	https://phenix-online.org/
COOT	Emsley et al., 2010	https://www2.mrc-lmb.cam.ac.uk/personal/pemsley/coot/
REFMAC5	Murshudov et al., 2011	https://www.ccp4.ac.uk/html/refmac5/description.html
PDB-REDO	Joosten et al., 2014	https://pdb-redo.eu
PyMOL	Schrödinger	https://pymol.org/2/
Adobe Illustrator	Adobe	https://www.adobe.com/uk/products/illustrator.html
MicroCal PEAQ-ITC	Malvern Panalytical	https://www.malvernpanalytical.com/en

**Other**		

HisTrap FF 5ml	Cytiva	Cat# 17525501
HiPrep 26/10 desalting	Cytiva	Cat# 17508701
Resource Q 6ml	Cytiva	Cat # 17117901
HiLoad 16/60 Superdex 200 pg	GE Healthcare	Cat# 28989335
HiLoad 16/60 Superdex 75 pg	Cytiva	Cat# 28989333
Odyssey CLx fluorescence imager	LI-COR	RRID: SCR_014579
NuPAGE™ 4 – 12% Bis-Tris SDS-PAGE gels	Thermo Scientific	Cat# NP0321; Cat# WG1402; Cat# WG1403A

### Resource availability

#### Lead contact

Information and requests for resources and reagents should be directed to the Lead Contact, Dr. Yogesh Kulathu (ykulathu@dundee.ac.uk).

#### Materials availability

Plasmids used in this study have been deposited with and will be distributed by MRC PPU reagents and services (https://mrcppureagents.dundee.ac.uk/). All unique/stable reagents generated in this study are available from the lead contact.

### Experimental model and subject details

#### BL21(DE3) *E. coli*

Proteins used for biochemistry and crystallography were expressed and purified from *E.coli* BL21(DE3) bacteria. Competent cells were stored at −80°C until use. Cells were grown in culture at 37°C with shaking until OD_600_ = 0.6-0.8. On induction of expression with IPTG, cells were grown overnight at 18°C with shaking.

### Method details

#### Plasmids

All cDNA constructs used in this study were generated by the Cloning team of the MRC reagents and services facility, MRC Protein Phosphorylation and Ubiquitylation Unit, University of Dundee, United Kingdom (see Table S3) and can be requested from MRC Reagents and Services (https://mrcppureagents.dundee.ac.uk/).

#### Protein expression and purification

All recombinant GST-fusion proteins were expressed in *E. coli* strain BL21(DE3). The bacterial cell cultures were grown in 2xTY media containing 100 μg/ml ampicillin to an OD_600_ of 0.6-0.8 at 37°C. Protein expression was induced with 300 μM IPTG followed by overnight shaking at 18°C. Cells were harvested at 4000 rpm for 15 minutes and the pellets were resuspended in GST-Lysis Buffer (50 mM Tris-HCl pH 7.5, 300 mM NaCl, 10% glycerol, 0.075% 2-mercaptoethanol, 1 mM benzamidine, 1 mM AEBSF, and complete protease inhibitor cocktail (Roche)). The resuspended cells were lysed by sonication and clarified by centrifugation at 30,000 x g for 45 min at 4°C and the lysates were incubated with Glutathione Sepharose 4B resin (Abcam) for 2 hr at 4°C on a rolling shaker. Resin was washed extensively, first with high salt buffer (25 mM Tris pH 7.5, 500 mM NaCl, and 10 mM DTT) and then with low salt buffer (50 mM Tris-HCl pH 7.5, 150 mM NaCl, 10% glycerol, and 1 mM DTT). The GST tag was removed by on column cleavage with 3C protease in an overnight incubation at 4°C. All purified proteins used for DUB assays or enzymes kinetics were quantified using nanodrop at A_280_ and aliquots were flashed frozen in liquid nitrogen and stored at −80°C. Proteins meant for ITC, SAXS or crystallization were further purified by anion exchange chromatography (Resource Q, GE Healthcare Life Sciences) and eluted in a gradient with buffer Q (50 mM Tris-HCl pH 8.5, 1 M NaCl and 2 mM DTT), followed by size exclusion chromatography (Superdex 75 16/60, GE Healthcare Life Sciences) in either: buffer I for ITC (50mM Tris-HCl pH 7.5, 150mM NaCl and 250μM TCEP), buffer S for SAXS (20 mM HEPES pH 7.5, 100 mM NaCl, 5mM DTT) or buffer X for crystallization (50 mM Tris-HCl, 150 mM NaCl, 10 mM DTT). The purified proteins were concentrated, quantified using nanodrop and flash frozen in liquid nitrogen and stored at −80°C.

#### Deubiquitylation assays using unlabelled polyUb chains

DUBs were diluted in 50 mM Tris-HCl pH 7.5, 50 mM NaCl, 10 mM DTT and incubated at room temperature (24°C) for 10 min to fully reduce the catalytic Cys. DUB assays were subsequently carried out where 1.9 μM of K48-Ub2 or K48-Ub3 were incubated with 1.6 μM of MINDY1 in 50 mM Tris-HCl pH 7.5, 50 mM NaCl, 10 mM DTT in a reaction volume of 10 μl. For DUB assay against different linkage types, 1.9 μM of diUb or 2.2 μM of tetraUb of specific linkage types were incubated with 1.6 μM of MINDY1 or 1.6 μM of MINDY2 in 50 mM Tris-HCl pH 7.5, 50 mM NaCl, 10 mM DTT in a reaction volume of 10 μL (Figures 2H and S1B). For DUB assays comparing activity of MINDY1^FL^ and MINDY1^cat^ at cleaving longer untaggedK48 chains, 3.5 μg of K48-Ub5-n or 2.2 μM of K48-Ub6 were incubated with 1.6 μM of MINDY1 in 50 mM Tris-HCl pH 7.5, 50 mM NaCl, 10 mM DTT in a reaction volume of 10 μl. For DUB assays comparing activity of MINDY2^FL^ and MINDY2^cat^ at cleaving longer untagged K48 chains, 3.5 μg of K48-Ub5-n were incubated with 0.1 μM of MINDY2 in 50 mM Tris-HCl pH 7.5, 50 mM NaCl, 10 mM DTT in a reaction volume of 10 μl.

For DUB assays comparing activity of MINDY2 S1, S1’, S2′, S3′ and S4’ site mutants and MINDY1 S2′, S3′ and S4’ site mutants at cleaving pentaUb, 100 nM MINDY1/2 was incubated with 1.25 μM K48-Ub5 in 50 mM Tris-HCl pH 7.5, 50 mM NaCl, 10 mM DTT. All reactions were incubated at 30°C and stopped at indicated time points by adding LDS buffer. The samples were separated on 4%–12% SDS-PAGE gel (Life Technology) and silver stained using Pierce Silver stain kit (Thermo Fisher).

#### Generating fluorescently labeled polyUb chains

Fluorescently labeled Ub5 was synthesized as described previously (Abdul Rehman et al., 2016). Briefly, Cys-Ub 1-75 (containing a Cys residue upstream of M1) was coupled to pre-formed Ub4. The cysteine residue of this proximal ubiquitin was then conjugated to IRDye-800CW (Li-Cor). Fluorescently labeled longer K48-linked chains (Ub_n_) were synthesized using a reaction mix containing 2500 μM WT ubiquitin, 250 μM (N-term 6His)-Ub (K48R, K63C), 0.5 μM UBE1, 15 μM UBE2R1, 10 mM ATP, 50 mM Tris-HCl (pH 7.5), 10 mM MgCl2, and 0.6 mM DTT with an overnight incubation at 30°C. Chains with successful incorporation of 6His-Ub (K48R, K63C) at their distal end were separated from WT chains using a HisTrap FF 5 mL column (Cytiva). Chains were then fractionated by size over a Superdex 200 16/60 column. Fractions containing chains between Ub7-Ub20 were pooled and concentrated and buffer exchanged into PBS. An approximate concentration was determined using the median chain length and the chains were then reacted with a 3-fold molar excess of IRDye-800CW (Li-Cor) for 3 h at 22°C (600 rpm). The reaction was quenched with 50 mM BME and excess dye removed by desalting with a Hiprep 26/10 desalting column (Cytiva) followed by size-exclusion chromatography with a Superdex 200 16/60 column (Cytiva).

#### Deubiquitylation assays using fluorescently labeled polyUb chains

Methods were the same for all fluorescent polyUb species used in this study. Both DUBs and fluorescently labeled K48-polyUb chains were diluted in 50 mM Tris-HCl pH 7.5, 50 mM NaCl, 10 mM DTT, and 0.25 mg/ml BSA. DUBs were activated by incubation at room temperature for 10 min. The reaction mixtures containing 1 μM DUB and 500 nM fluorescently labeled K48-linked chains were incubated at 30°C (5 μM of MINDY1^Cat/FL^ was used in assays with longer chains, 100 nM MINDY2 was used in the assay comparing MINDY2 WT with ubiquitin binding site mutants). At the indicated time points, 2.5 μl of the samples was transferred to 7.5 μl LDS sample buffer to quench the reaction. The samples were resolved on 4%–12% Bist-Tris SDS gels (NuPAGE, Thermofisher) and the gels were scanned with Odyssey® CLx Imaging System at 800 nm channel and quantified with Image Studio Lite software. Data from two independent experiments were fitted using nonlinear regression. Data fitting was performed using GraphPad Prism 8 software.

#### Enzyme kinetics

Steady-state kinetics of K48 linked Ub5 fluorescent chain (IR-K48-Ub5) hydrolysis by MINDY1, MINDY2 and their mutants (Y114A^MINDY1^, P138A^MINDY1^, Y243A^MINDY2^ and P267A^MINDY2^). The catalytic domain of MINDY1 and MINDY2 and their mutants were incubated with varying concentrations of K48 linked Ub5 fluorescent chains and the formation of K48 Ub4 at the early time points was quantified to obtain the initial velocities. Both DUBs and fluorescently labeled K48-polyUb chains were diluted in 50 mM Tris-HCl pH 7.5, 50 mM NaCl, 10 mM DTT, and 0.25 mg/ml BSA. At the indicated time points (0, 3, 6, 9 and 12 minutes), 2.5 μl of the samples was transferred to 7.5 μl LDS sample buffer to quench the reaction. The SDS gels of DUB assays were scanned with Odyssey® CLx Imaging System at 800 nm channel and quantified with Image Studio Lite software. The amount of K48-Ub4 formed was plotted against time and the data was fitted to a linear regression curve, where slope is the initial velocity, V_o_ (M.s-1). The initial velocities obtained were plotted against the of K48 linked Ub5 fluorescent chains (substrate) concentration and the curves were fitted to Michaelis-Menten equation to estimate the K_cat_ and K_m_ (n = 2; mean ± SD). Data fitting was carried out using GraphPad Prism 8 software.

#### ITC measurements

ITC measurements were performed on MicroCal PEAQ-ITC (Malvern) at 25°C. Prior to measurements, all proteins were dialysed into a buffer containing 50mM Tris-HCl pH 7.5, 150mM NaCl and 250μM TCEP. The syringe contained MINDY1 and was titrated into the cell which contained K48-linked polyubiquitin chains (K48-diUb, triUb or tetraUb or pentaUb). 2 μl of MINDY1 was dispensed in 4 s duration with 130 s spacing in between injections for a total of 16 injections. Data were analyzed and titration curves were fitted using MicroCal PEAQ-ITC (Malvern) analysis software (n = 2; mean ± SD).

#### Crystallization and structure determination

##### MINDY1^C137A^:K48-Ub2

The MINDY1-catalytic domain C137A mutant construct (residues 110-384) in 50 mM Tris-HCl pH 7.5, 150 mM NaCl, and 10 mM DTT was mixed with K48-linked diUb in a 1:1 ratio and concentrated to a final concentration of 11.5 mg/ml. The crystals were grown in hanging drop 24 well plates against the well solution of 0.1 M Tris-HCl pH 8.5, 0.2 M Lithium sulfate monohydrate and 30% PEG 400. The crystals were flash frozen in cryo-protectant containing 0.1 M Tris-HCl pH 8.5, 0.2 M Lithium sulfate monohydrate and 35% PEG 400. Diffraction data were collected at ID23-1 beamline, ESRF, France (wavelength 0.9397 Å). The datasets were processed using XDS (Kabsch, 2010) and then scaled using AIMLESS (Evans and Murshudov, 2013). The structure of the complex MINDY1^C137A^:K48-Ub2 was solved by molecular replacement (Phaser) (McCoy et al., 2007) using MINDY1^apo^ (PDB ID: 5JKN) and Ub (PDB ID: 1UBQ) as search models. The partially built model obtained was further manually built in COOT. The complete model was obtained after iterative building and refinement with COOT (Emsley et al., 2010) and REFAMC5 (Murshudov et al., 2011). The final structure was re-refined using PDB-REDO (Joosten et al., 2014). The final data collection and refinement statistics for the MINDY1^C137A^:K48-Ub2 complex structure is shown in Table S4. All Figs were made using PyMOL (https://pymol.org/2/).

##### MINDY2^C266A^:K48-Ub2

The MINDY2-catalytic domain C266A mutant construct (residues 241-504) was expressed in *E.coli* BL21(DE3) cells as described. Purified MINDY2 protein in 50 mM Tris-HCl pH 7.5, 150 mM NaCl, and 10 mM DTT was mixed with K48-linked diUb in a 1:1 ratio and concentrated to a final concentration of 18.0 mg/ml. The crystals grew grew in hanging drops (24 well plates) from conditions with 0.05 M Potassium phosphate monobasic and 20% PEG 8000. The crystals were flash frozen in cryoprotectant containing 0.05 M Potassium phosphate monobasic and 5% PEG 8000 and 30% PEG 8000. Diffraction data were collected at ID29 beamline, ESRF, France (wavelength 0.97625 Å). The datasets were processed, and structures determined as for MINDY1^C137A^:K48-Ub2 structure.

##### P138AC137A:K48-Ub2

The MINDY1 P138A C137A mutant construct (residues 110-384) was expressed in *E. coli* BL21(DE3) cells and purified as described. Purified protein in 50 mM Tris-HCl pH 7.5, 150 mM NaCl, and 10 mM DTT was mixed with K48-linked diUb in a 1:1 ratio and concentrated to a final concentration of 14.0 mg/ml. The crystals were grown in hanging drop 24 well plates against the well solution of 0.2 M sodium malonate pH 7.0 and 20% PEG 3350. The crystals were flash frozen in cryo-protectant containing 0.2 M sodium malonate pH 7.0 and 35% PEG 400. Diffraction data were collected at ID30B beamline, ESRF, France (wavelength 0.99187 Å).The datasets were processed, and structures determined as for MINDY1^C137A^:K48-Ub2 structure.

##### MINDY2^apo^

The catalytic domain of MINDY2 (residues 241-504) was expressed in *E.coli* BL21(DE3) cells and purified as described. MINDY2^apo^ protein in 50 mM Tris-HCl pH 7.5, 150 mM NaCl, and 10 mM DTT. The crystals were grown from hanging drops containing an equal volume of protein (12.5 mg/ml) and mother liquor containing 0.1 M Bis Tris pH 6.0, 0.2 M MgCl_2_ and 25% PEG 3350. The crystals were flash frozen in cryoprotectant containing 0.1 M Bis Tris pH 6.0, 0.2 M MgCl_2_ and 35% PEG 400. Diffraction data were collected at ID29 beamline, ESRF, France (wavelength 1.07252 Å). The datasets were processed using XDS (Kabsch, 2010) and then scaled using AIMLESS (Evans and Murshudov, 2013). The structure of the MINDY2^apo^ was solved by molecular replacement (MoRDa) using MINDY1^apo^ (PDB ID: 5JKN) as search model.

##### MINDY1 Y114F

The catalytic domain of MINDY1 Y114F construct (residues 110-384) was expressed and purified as described. The crystals were grown from hanging drops containing an equal volume of protein (11.5 mg/ml) and mother liquor containing 0.1 M Tris-HCl pH 8.5, 1.5 M Ammonium phosphate dibasic. The crystals were flash frozen in cryoprotectant containing 0.1 M Tris-HCl pH 8.5, 3.4 M Sodium malonate pH 8.0 and 20% glycerol. Diffraction data were collected at ID23-2 beamline, ESRF, France (wavelength 0.87313 Å). The structure of the Y114F was solved by molecular replacement (Phaser) using MINDY1^apo^ (PDB ID: 5JKN) as search model.

##### MINDY1 P138A

MINDY1 P138A (residues 110-384) was expressed and purified as described. The crystals were grown from hanging drops containing an equal volume of protein (11.05 mg/ml) and mother liquor containing 0.1 M Bis Tris Propane pH 7.0 and 0.7 M sodium citrate tribasic dihydrate. The crystals were flash frozen in cryoprotectant containing 0.1 M Bis Tris Propane pH 7.0 and 0.7 M sodium citrate tribasic dihydrate and 30% PEG 400. Diffraction data were collected at ID23-1 beamline, ESRF, France (wavelength 0.97625 Å). The structure of the P138A was solved by molecular replacement (Phaser) using MINDY1^apo^ (PDB ID: 5JKN) as search model.

##### MINDY1 T335D

MINDY1 T335D (residues 110-384) was expressed and purified as described. The crystals were grown from hanging drops containing an equal volume of protein (12.0 mg/ml) and mother liquor containing 0.1 M HEPES-Na pH 7.5 and 0.8 M Potassium sodium tartrate tetrahydrate. The crystals were flash frozen in cryoprotectant containing 0.07 M HEPES-Na pH 7.5, 0.52 M Potassium sodium tartrate tetrahydrate and 35% glycerol. Diffraction data were collected at I03 beamline, Diamond, UK (wavelength 0.97628 Å). The structure of the T335D was solved by molecular replacement (Phaser) using MINDY1^apo^ (PDB ID: 5JKN) as search model.

##### MINDY2^C266A^:K48-Ub5

The catalytic domain of MINDY2^C266A^ (residues 241-504) was expressed in *E.coli* BL21(DE3) cells and purified as previously described. Purified protein in 50 mM Tris-HCl pH 7.5, 150 mM NaCl, and 10 mM DTT was mixed with K48-linked Ub5 in a 1:1 ratio and concentrated to a final concentration of 13.5 mg/ml. Crystals were grown in hanging drops containing a 2-fold excess of protein over mother liquor containing 3% (w/v) dextran sulfate sodium salt, 0.1 M BICINE pH 8.5 and 15% (w/v) PEG 20,000. Crystals were frozen in cryoprotectant consisting of mother liquor supplemented with 30% ethylene glycol. Diffraction data were collected at ID30-A-1 beamline, ESRF, France. The structure was solved by molecular replacement (Phaser) using MINDY2^C266A^:K48-Ub2 as a search model. The asymmetric unit contained 7 copies of the complex and clear electron density was visible for MINDY2 and the Ub molecules bound at the S1, S1’, S3′ and S4’ sites. While large regions of the Ub at the S2′ site are poorly represented in the electron density, well defined electron density of the β3- and β5-strands, as well as the C terminus of this ubiquitin molecule allowed precise positioning of this entity.

#### Sequence conservation

Sequences of metazoan orthologs of MINDY1 (OMA Group 804311) and MINDY2 (OMA Group 574560) were retrieved from the OMA Orthology database (https://omabrowser.org/) (Altenhoff et al., 2021). The orthologous sequences of each MINDY were aligned using the Clustal Omega Multiple Sequence Alignment Tool (https://www.ebi.ac.uk/Tools/msa/clustalo/) (Madeira et al., 2019). The sequence alignments were used for surface conservation analysis with chain A of MINDY1 (PDB 6TUV) and MINDY2 (PDB 7NPI), respectively, using the Consurf webserver (https://consurf.tau.ac.il/) (Ashkenazy et al., 2016).

#### Small angle X-ray scattering (SAXS)

Synchrotron radiation X-ray scattering patterns from MINDY2-apo, in complex with Ub^Prg^, and polyubiquitin were collected at the EMBL P12 beamline of the storage ring PETRA III (DESY, Hamburg, Germany) (Blanchet et al., 2015). Images were recorded using a photon counting Pilatus-6M detector at a sample to detector distance of 3.0 m and a wavelength (λ) of 0.12 nm covering the range of momentum transfer 0.01 < s < 7 nm^-1^ with s = 4πsinθ/λ, where 2θ is the scattering angle. To obtain data from monodisperse samples (MINDY2-apo, MINDY2∼Ub, MINDY2-Ub2, MINDY2-Ub3, MINDY2-Ub4 and MINDY2-Ub5), samples were passed through size exclusion chromatography (Superdex 200 10/300) directly coupled to the SAXS instrument (SEC-SAXS). Only frames corresponding to the main elution peaks were considered. The SAXS data collected from the buffer components before the elution peak was used for background subtraction. The overall structural parameters of the apo construct derived from the SAXS data are compatible with a monomeric species while those for the complexes show trends typical of complex formation and thus confirm the binding of the increasing number of ubiquitin molecules to MINDY2 (Table S2).

One second sample exposures were recorded throughout the entire chromatography step. Buffer S (20 mM HEPES pH 7.5, 100 mM NaCl, 5mM DTT) was used as mobile phase. 100 μl of purified sample were injected onto a Superdex 200 10/300 (GE Healthcare) column and the flow rate was set to 0.5 ml/min. SAXS data were also recorded from macromolecule-free fractions corresponding to the matched solvent blank. Data reduction to produce final scattering profiles of MINDY 2 constructs was performed using standard methods. Briefly, 2D-to-1D radial averaging was performed by the SASFLOW pipeline (Franke et al., 2017) CHROMIXS was used for visualization and reduction of the SEC-SAXS datasets (Panjkovich and Svergun, 2018). Aided by the integrated prediction algorithms in CHROMIXS the optimal frames within the elution peak and the buffer regions were selected. Single buffer frames were then subtracted from sample frames one by one, scaled and averaged to produce the final subtracted curve. The radius of gyration R_G_ was computed for each construct by Guinier approximation (Guinier, 1939). The molecular mass (MM) of the solutes was evaluated based on the concentration independent approach using Porod invariant (Porod, 1951) as implemented in the ATSAS package (Hajizadeh et al., 2018). The indirect Fourier transform of the SAXS data and the corresponding probable real space pair distance distribution (p(r) versus r profile) of the MINDY2 constructs were calculated using GNOM (Svergun, 1992) yielding also the particle diameter D_max_. The theoretical curves were calculated from the atomic models with CRYSOL (Svergun et al., 1995). In case of systematic deviations, normal mode analysis as implemented in SREFLEX (Panjkovich and Svergun, 2016) was employed to refine the crystallographic models. Possibilities of having mixtures of complexes with distinguishable Ub2 binding positions were analyzed with OLIGOMER (Konarev et al., 2003).The SAXS data (as summarized in Table S2) as well as fits to the curves computed from the crystal structures and from the refined models have been deposited into the Small-Angle Scattering Biological Data Bank (SASBDB) (Valentini et al., 2015).

### Quantification and statistical analysis

All gels using fluorescent chains were repeated such that n = 3. Intensities were quantified using ImageStudioLite (Li-Cor). Intensities were input into GraphPad Prism 8.2.1 from which SD/SEM were derived and graphs plotted. All kinetic parameters were determined as described and DUB assays quantified using GraphPad Prism 8.2.1 software. ITC data were analyzed and titration curves were fitted using MicroCal PEAQ-ITC (Malvern) analysis software (n = 2; mean ± SD).

## Data Availability

• All crystallographic and small-angle scattering data have been deposited in the PDB and SASBDB, respectively, and are publicly available as of the date of publication. Accession numbers are listed in the Key resources table. Original gel scans have been deposited at Mendeley and are publicly available as of the date of publication. The DOI is listed in the Key resources table. • This paper does not report original code • Any additional information required to reanalyze the data reported in this paper is available from the lead contact upon request.

## Supplemental information

Document S1. Figures S1–S8 and Tables S1–S3

Table S4. Crystallography data collection and refinement statistics

Document S2. Article plus supplemental information
