# Supplementary material for: Mechanism of activation and regulation of deubiquitinase activity in MINDY1 and MINDY2
Source: Mol Cell. 2021 Oct 21;81(20):4176–4190.e6. doi: 10.1016/j.molcel.2021.08.024 (PMC8550791; doi:10.1016/j.molcel.2021.08.024)
Supplement: Document S1. Figures S1–S8 and Tables S1–S3 [file mmc1.pdf]

Molecular Cell, Volume *81*

Supplemental information

Mechanism of activation and regulation of deubiquitinase activity in MINDY1 and MINDY2

Syed Arif Abdul Rehman, Lee A. Armstrong, Sven M. Lange, Yosua Adi Kristariyanto, Tobias W. Gräwert, Axel Knebel, Dmitri I. Svergun, and Yogesh Kulathu

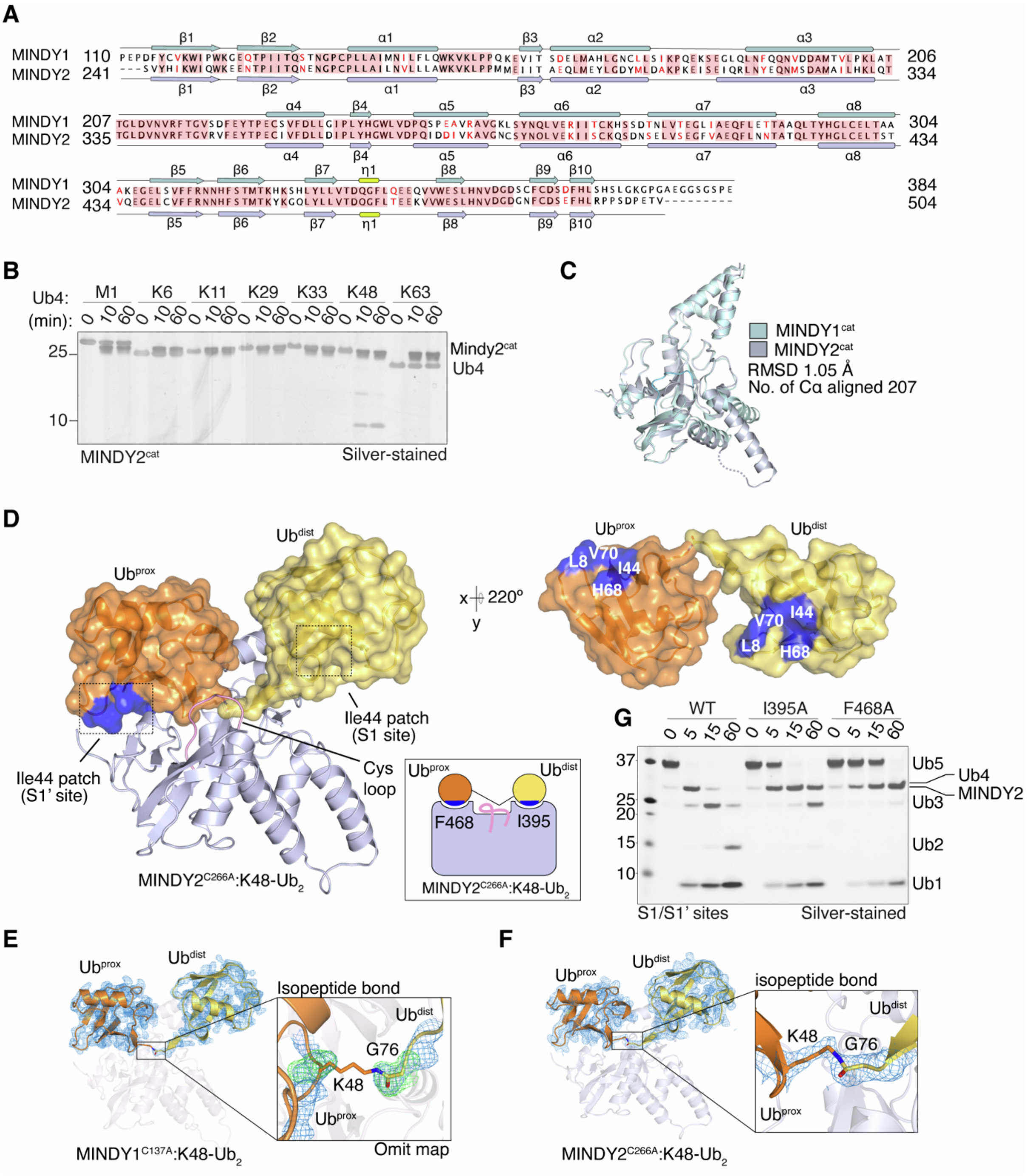

Figure S1 Crystal structure of MINDY1/2 in complex with K48-linked diUb

A) Secondary structure alignment of MINDY1 and MINDY2 based on their crystal structures using ESPRIPT webserver (http://espript.ibcp.fr/ESPript/ESPript/). The secondary structure elements of MINDY1 and MINDY2 are shown in pale cyan and light orange respectively.

B) Silver stained gels of DUB assays testing activity and specificity of polyUb cleavage by MINDY2 catalytic domain (241–504). 1.6 μM of DUB was incubated with 2.2 μM of tetraUb chains for the indicated time points.

C) Superposition of the crystal structures of the minimal catalytic domains of MINDY1 (pale-cyan) and MINDY2 (light-orange) shown in cartoon representation.

D) The MINDY2^{C266A}:K48-diUb complex crystal structure is shown with MINDY2 in cartoon (blue white). Ub molecules are depicted with transparent surfaces (tv-orange:Ub^{prox} and yelloworange: Ub^{dist}). I44 patches on Ub are coloured blue and an alternate view of the bound diUb rotated by 220° along the x-axis is shown on the right side. Schematic representation of MINDY2^{C266A}:K48-diUb complex (inset).

E) The crystal structure of MINDY1^{C137A}:K48-diUb complex with electron density for both proximal and distal Ub (orange and light-brown) contoured at 1.0 σ. In the inset, the Fo – Fc omit electron density map was generated from coefficients calculated by removing G76 and K48 from the distal and proximal Ub moieties, respectively, from the atomic model for 10 cycles of refinement. The Fo−Fc map is contoured at 3.0 σ whereas 2Fo−Fc map is contoured at 1.0 σ.

F) As in (E) for the MINDY2^{C266A}:K48-diUb complex.

G) DUB assay monitoring cleavage of K48-Ub_5 by MINDY2^{cat} and the indicated S1 and S1' mutants.

Related to Figure 1

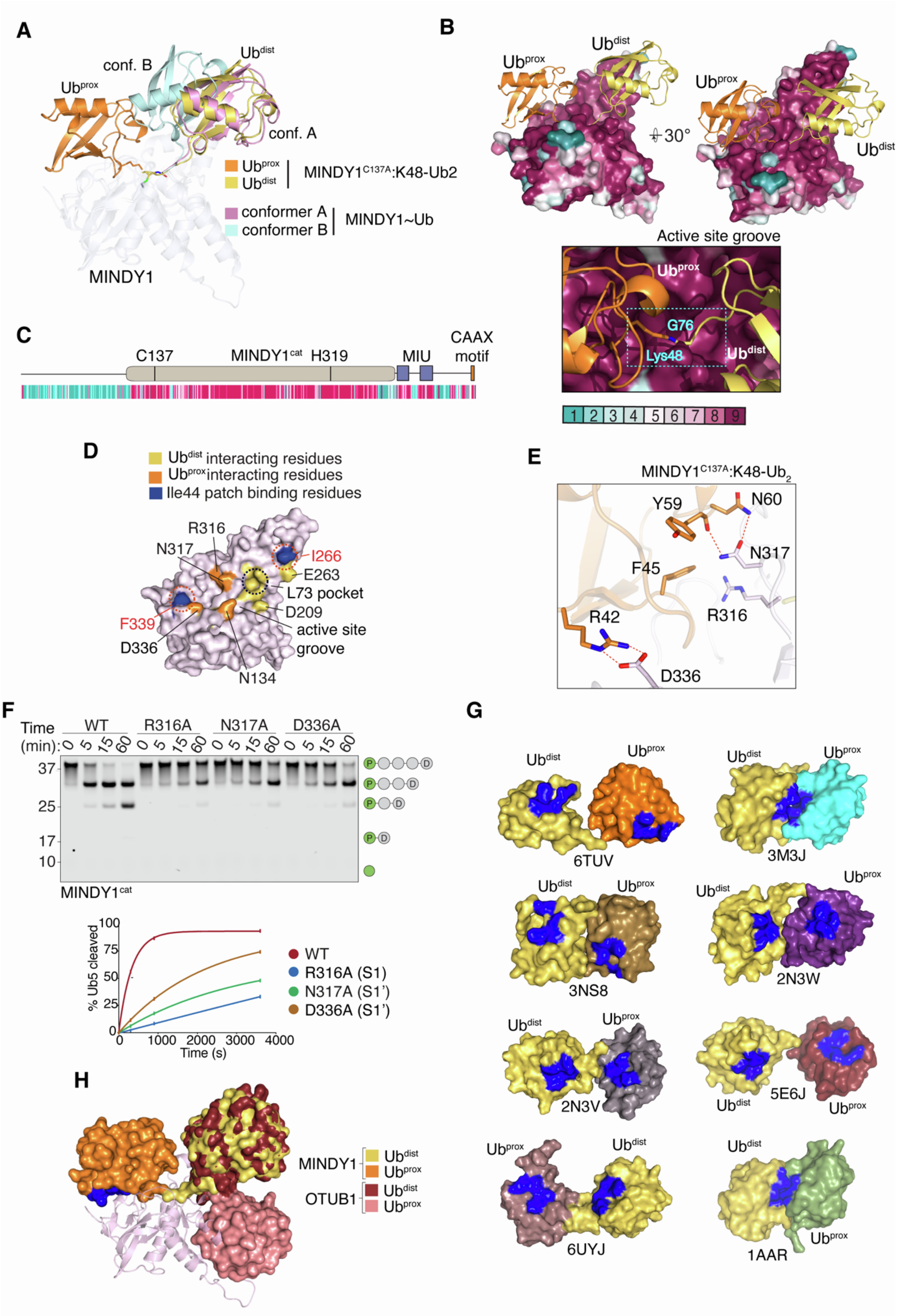

Figure S2 Analysis of K48-Ub2 interaction with MINDY1

A) Superposition of the MINDY1^{C137A}:K48-Ub2 complex with MINDY1~Ub^{Prg} complex reveals that proximal Ub stabilises the binding of Ub onto the S1 site.

B-C) The evolutionary conservation score of MINDY1 was calculated using the crystal structure of MINDY1 and primary sequences from 19 species using the ConSurf webserver. The conservation score was projected both on to the primary sequence (A) and the surface representation of the crystal structure of MINDY1 (panel B) The distal and proximal Ub binding region has been highlighted. In the inset, a close-up view reveals conservation of the active site and the catalytic groove (cyan dashed box) accommodating the scissile bond across the active site.

D) Surface representation of MINDY1 highlighting key interaction interfaces.

E) Close-up view of the critical polar interaction between MINDY1 (pink) and the proximal Ub (orange).

F) DUB assay comparing activity of different S1' site mutants at cleaving fluorescently-labelled pentaUb. The percent hydrolysis of K48 linked polyUb chains for the different mutants is plotted against time for the DUB assay (bottom).

G) Comparison of different K48-linked diUb structures shown in surface representation with the I44 patches highlighted in blue. MINDY1^{C137A}:K48-diUb (PDB ID: 6TUV); Solution structure of the Rpn1 T1 site with K48-linked diUb in the contracted binding mode (PDB ID: 2N3W) and in the extended binding mode (PDB ID: 2N3V) (Chen et al., 2016); A new crystal form of K48-linked diUb (PDB ID: 3M3J) (Trempe et al., 2010); Crystal structure of an open conformation of K48-linked diUb at pH 7.5 (PDB ID: 3NS8) ; Crystal structure of diUb bound to SARS PLpro (PDBID: 5E6J) (Békés et al., 2016b); hRpn13:hRpn2:K48-diUb structure (PDB ID: 6UYJ) (Lu et al., 2020) and crystal structure of a diUb and model for interaction with E2 (PDBID:1AAR) (Cook et al., 1992b).

H) Comparison of the conformation adopted by the Ub molecules present in the OTUB1~Ub:Ubc13~Ub complex (PDB ID: 4LDT) superposed on the distal Ub of MINDY1^{C137A}:K48-diUb (PDB ID: 6TUV)

Related to Figure 1

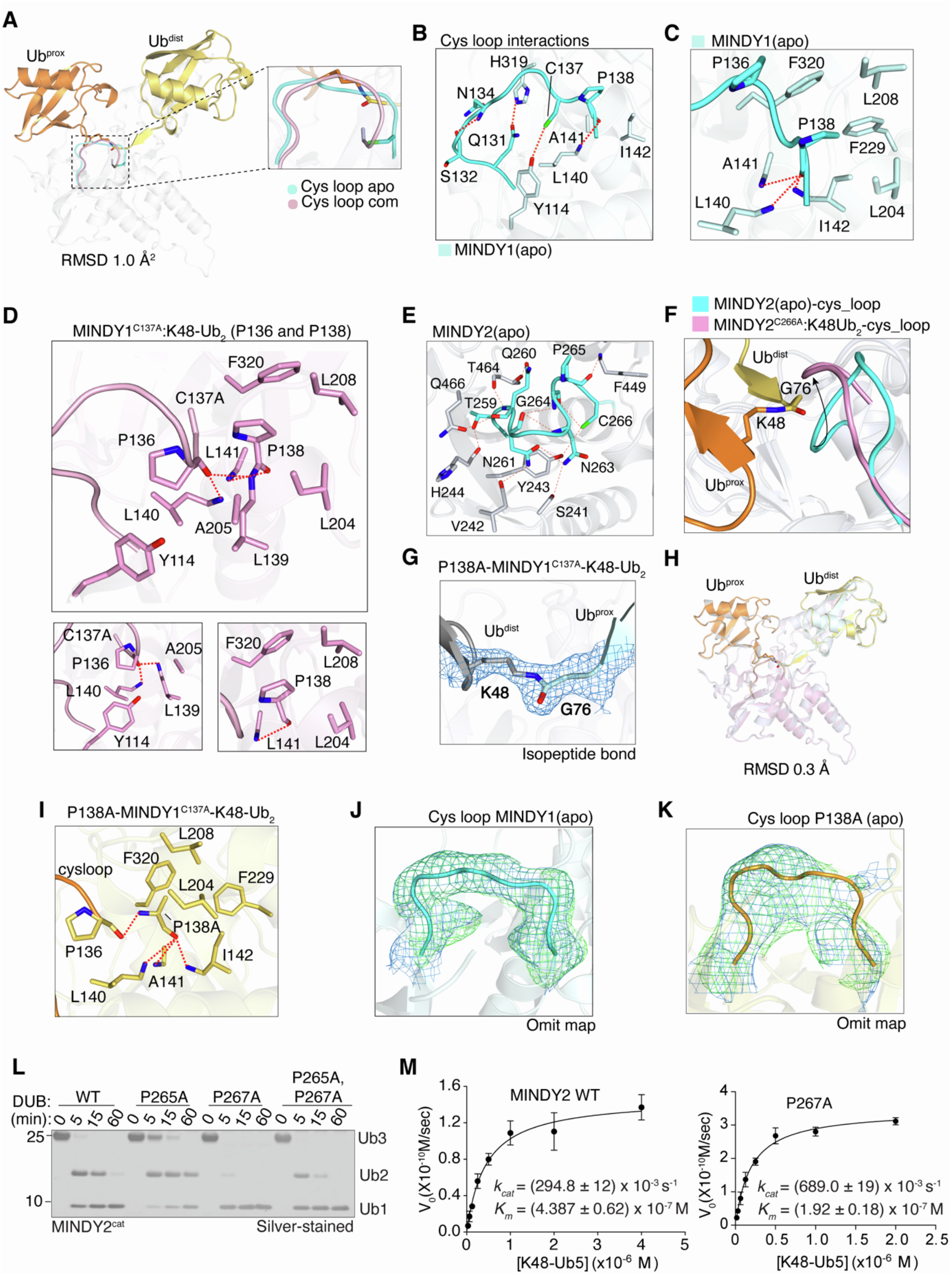

Figure S3 Cys loop regulates activity of MINDY1 and MINDY2

A) Cartoon representation of the crystal structures of MINDY1(apo) and the MINDY1^{C137A}:K48-Ub$_2$ complexes (RMSD 1.0 Å). Close up view of the Cys loop in the apo state (cyan) and active state (pink).

B) The hydrogen bond network of the Cys loop of MINDY1(apo). Dashed lines indicate hydrogen bonds.

C) Hydrogen bonds formed by the backbone of P138 in MINDY1 (apo).

D) Close up views of hydrophobic interactions (left) and hydrogen bonding with P136 and P138 (right) in the $MINDY1^{C137A}$:K48-Ub_2 complex.

E) Interaction network of the Cys loop in MINDY2 apo.

F) Superposition of the Cys loop in $MINDY2^{apo}$ (cyan) and $MINDY2^{C266A}$:K48-Ub_2 complex (pink). The incoming isopeptide can be seen clashing with the Cys loop in MINDY2 apo.

G) Close-up view showing electron density *2Fo-Fc* map contoured at 1.0 s of the isopeptide bond of the K48-Ub_2 from the P138A:K48-Ub_2 complex.

H) Superposition of $MINDY1^{C137A}$:K48-Ub_2 and $MINDY1^{C137A\ P138A}$:K48-Ub_2 complexes.

I) Close-up view of the $MINDY1^{C137A\ P138A}$:K48-Ub_2 complex showing the hydrogen bonding network of the mutant Cys loop.

J-K) Omit maps for $MINDY1^{apo}$ and MINDY1 P138A Cys loops. The $|F\text{o}|-|F\text{c}|$ electron density map was generated from coefficients calculated by deleting the Cys loop residues from the respective atomic models for 10 cycles of refinement. The $F\text{o}-F\text{c}$ map is contoured at 3.0 σ and the $2F\text{o}-F\text{c}$ map is contoured at 1.0 σ.

L) DUB assay monitoring K48-Ub_3 chain hydrolysis by MINDY2 WT and the mutants P265A, P267A and the double mutant P265A P267A. These two prolines flank the catalytic cysteine (C266).

M) Steady-state enzyme kinetics of K48-linked pentaUb cleavage by MINDY2 WT and the P267A mutant. The DUB was incubated with varying concentrations of fluorescently labelled pentaUb. (n= 2; mean ± SD).

Related to Figure 2

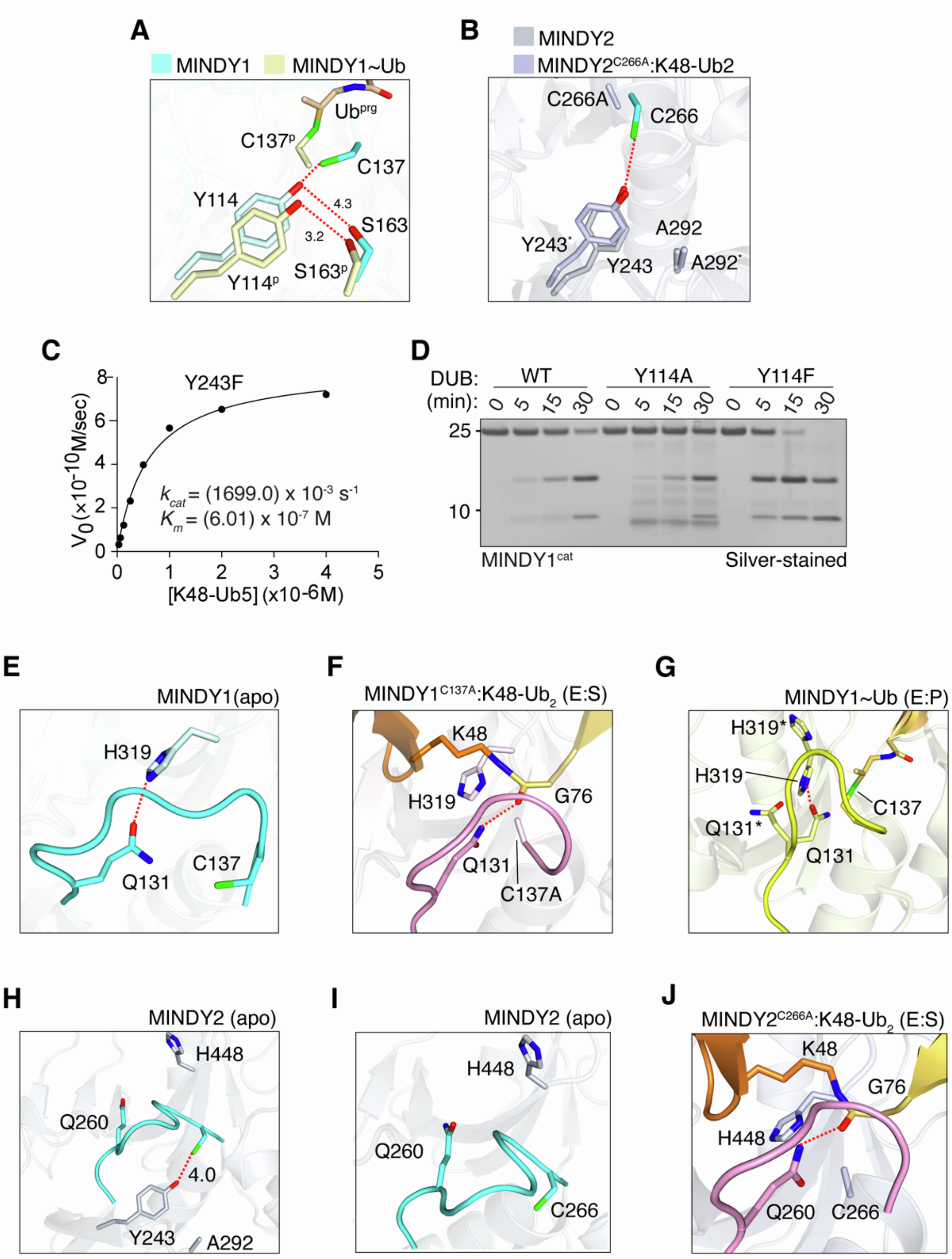

Figure S4 Autoinhibition and activation of MINDY1 and MINDY2

A) Close up view of Y114 in a superposition between MINDY1 (apo) and MINDY1~Ub^{Prg}. Dotted lines indicate hydrogen bond.

B) Close up view of superposition of MINDY2 apo and $MINDY2^{C266A}$:K48-Ub2 complex showing Y243. A292, the equivalent residue of S163 in MINDY2 does not induce lateral movement of the tyrosine observed in MINDY1. Asterisk indicates residues in complex.

C) Steady-state enzyme kinetics of K48-linked pentaUb cleavage by MINDY2 Y243F mutant. (n=1)

D) DUB assay monitoring the cleavage of K48linked-triUb chain by MINDY1 WT and Y114A and Y114F mutants. Of note, expression and stability of MINDY1 is affected by the Y114A mutation suggesting an additional structural role for Y114 in stabilizing local structure especially around the Cys loop.

E) A close-up view of MINDY1 apo catalytic site. The catalytic cysteine (C137) is rotated away from hydrogen bonding distance with the catalytic histidine (H319). Dotted lines indicate hydrogen bond.

F) A close-up view of the catalytic site in the MINDY1^{C137A}:K48-Ub$_2$ complex. The isopeptide bond can be seen interacting with the catalytic H319. Q131 is seen interacting with the isopeptide bond. Dotted lines indicate hydrogen bond.

G) A close-up view of the catalytic site in the MINDY1~UbPrg complex, where both H319 and Q131 exist in two alternate conformations. Dotted lines indicate hydrogen bond.

H) A close-up view of the catalytic site in MINDY2 showing key residues. The catalytic cysteine (C266) in MINDY2 is out of plane with the other catalytic residues and instead can be seen interacting with the non-catalytic Y243. Unlike MINDY1, the oxyanion forming glutamine (Q260) is not able to form any bonds with the flipped out catalytic histidine (H448). Dashed lines indicate hydrogen bond.

I) A close-up view of MINDY2 (apo) showing the catalytic cysteine (C266) rotated away from the hydrogen bonding distance with the catalytic histidine (H448).

J) View of the catalytic site in the MINDY2^{C266A}:K48-Ub$_2$ complex showing catalytically productive rearrangements upon K48-diUb binding. The catalytic histidine (H448) is seen to have flipped in plane with the catalytic cysteine (C266*). The oxyanion forming glutamine (Q260) is seen interacting with the isopeptide bond of the bound K48 chain.

Related to Figure 3

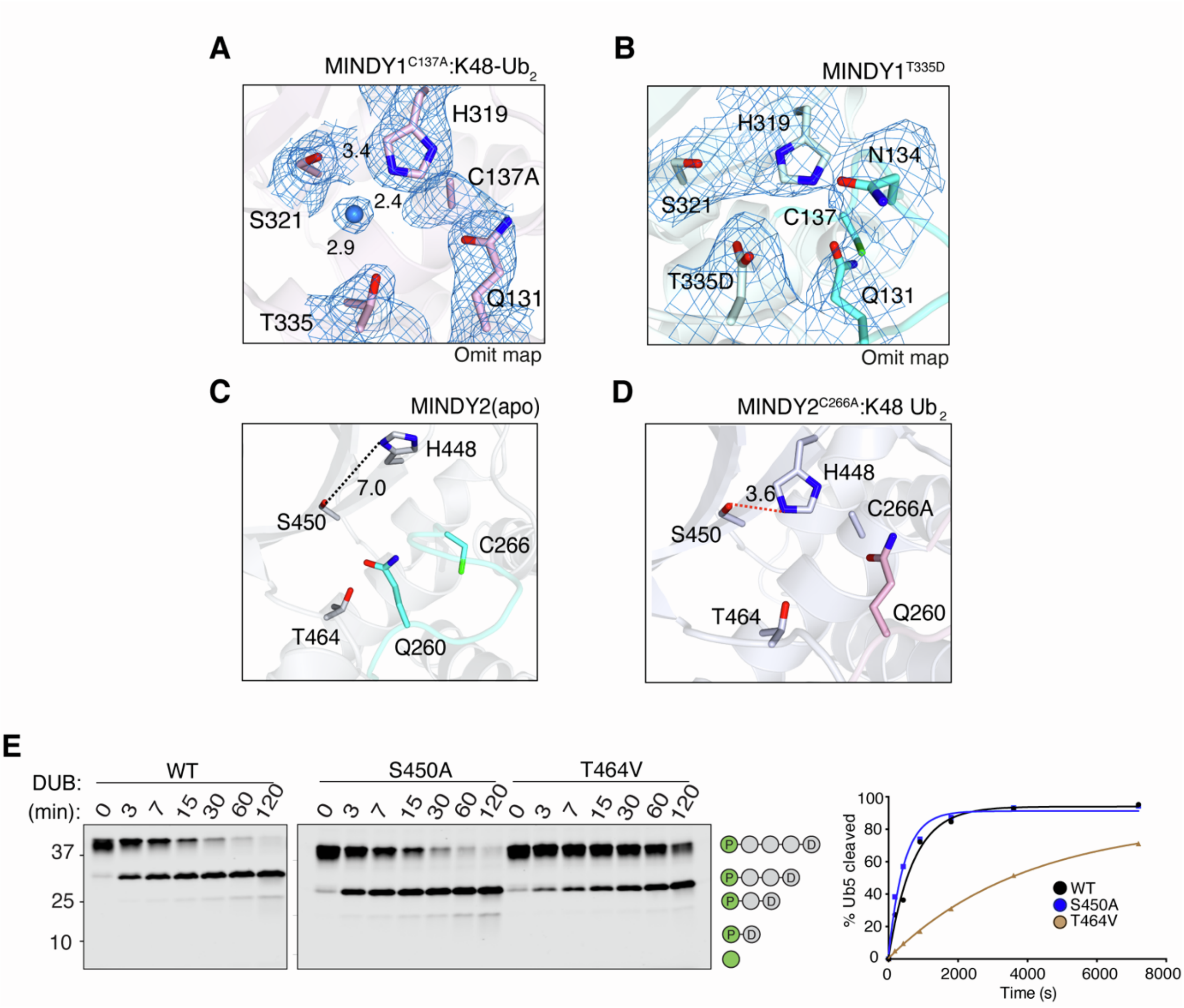

Figure S5 Catalytic mechanism of MINDY2

A) Close up view of the catalytic site in the crystal structure of MINDY1^{C137A}:K48-Ub2 showing the coordination of a water molecule by T335 and H319. The respective residues are shown with *2Fo-Fc* electron density contoured at 1.0 σ.

B) Close up view of the catalytic site in the crystal structure of MINDY1 T335D showing the formation of an ionic bond between T335D and the catalytic histidine (H319). The respective residues are shown with *2Fo-Fc* electron density contoured at 1.0 σ.

C)- **D**) Close up view of the catalytic site architecture in inhibited state MINDY2 apo (B) and in the active state in MINDY2^{C266A}:K48-Ub$_2$ (C).

E) DUB assay monitoring cleavage of fluorescently labelled K48-linked pentaUb chains by MINDY2 and the indicated mutants. Quantification of pentaUb cleavage is shown on the right. Gel is truncated to exclude mutants irrelevant to the study.

Related to Figure 4

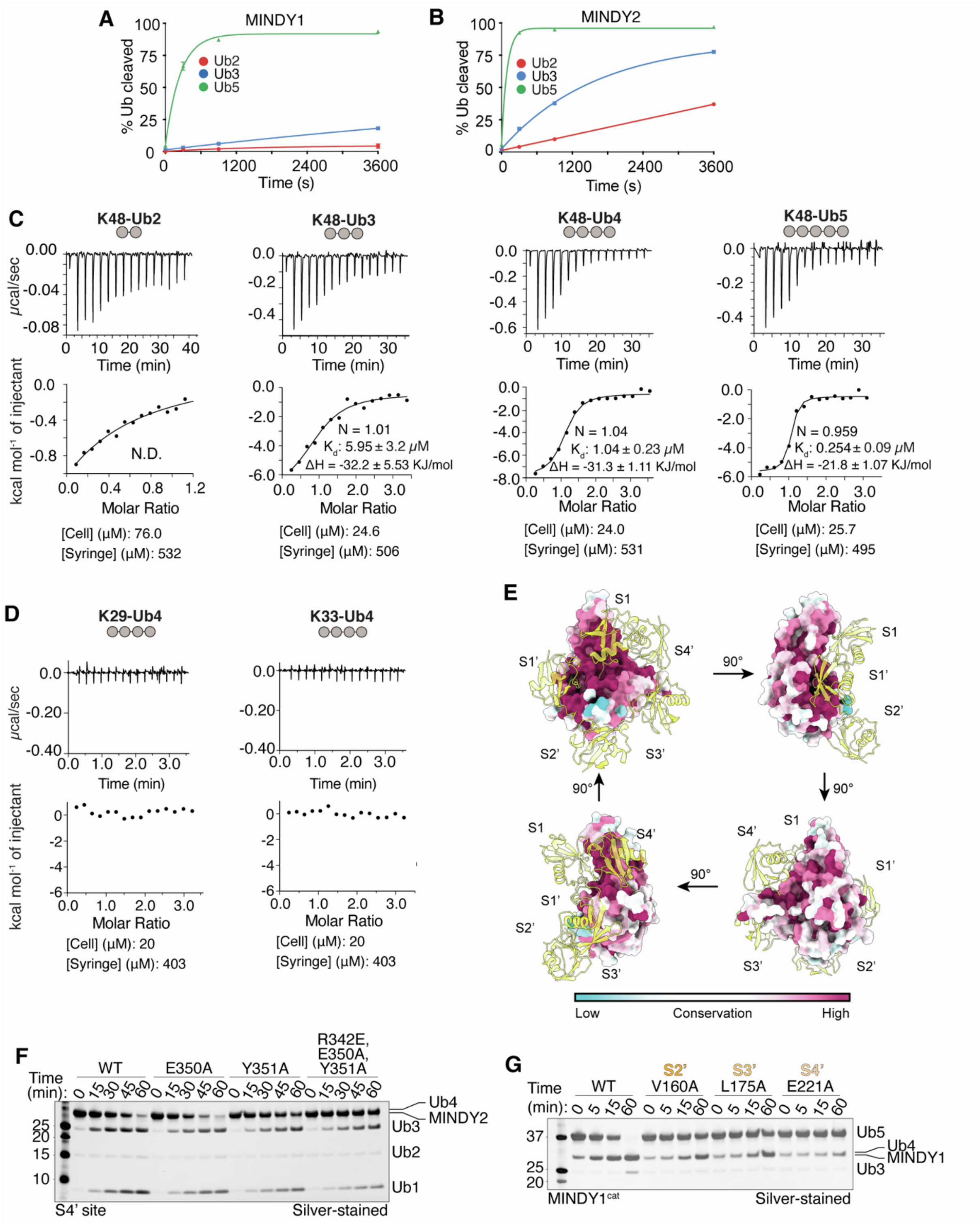

Figure S6 Recognition of K48 chains by MINDY1/2

A-B) MINDY1 and MINDY2 prefer to cleave longer chains. The percentage of fluorescently labelled K48-linked Ub_2, Ub_3, and Ub_5 cleaved over time by MINDY1 and MINDY2 is plotted. n=2; mean±SD

C) Isothermal titratison calorimetry (ITC) data measuring binding of MINDY1^{C137A} with K48-linked polyUb chains of the indicated lengths. Indicated concentrations of MINDY in the syringe was injected into polyUb chains in the cell. n=2; mean±SD

D) ITC data measuring binding of MINDY1^{C137A} to K29- and K33-linked tetraUb. Indicated concentrations of MINDY in the syringe was injected into tetraUb chains in the cell.

E) Consurf analysis of the Ub binding sites on MINDY1

F) DUB assay of a series of mutations at the S4' site of MINDY2 against K48-Ub4

G) DUB assay comparing cleavage of K48-Ub5 by WT MINDY1^{cat} and S2', S3' and S4' site mutants.

Related to Figure 5

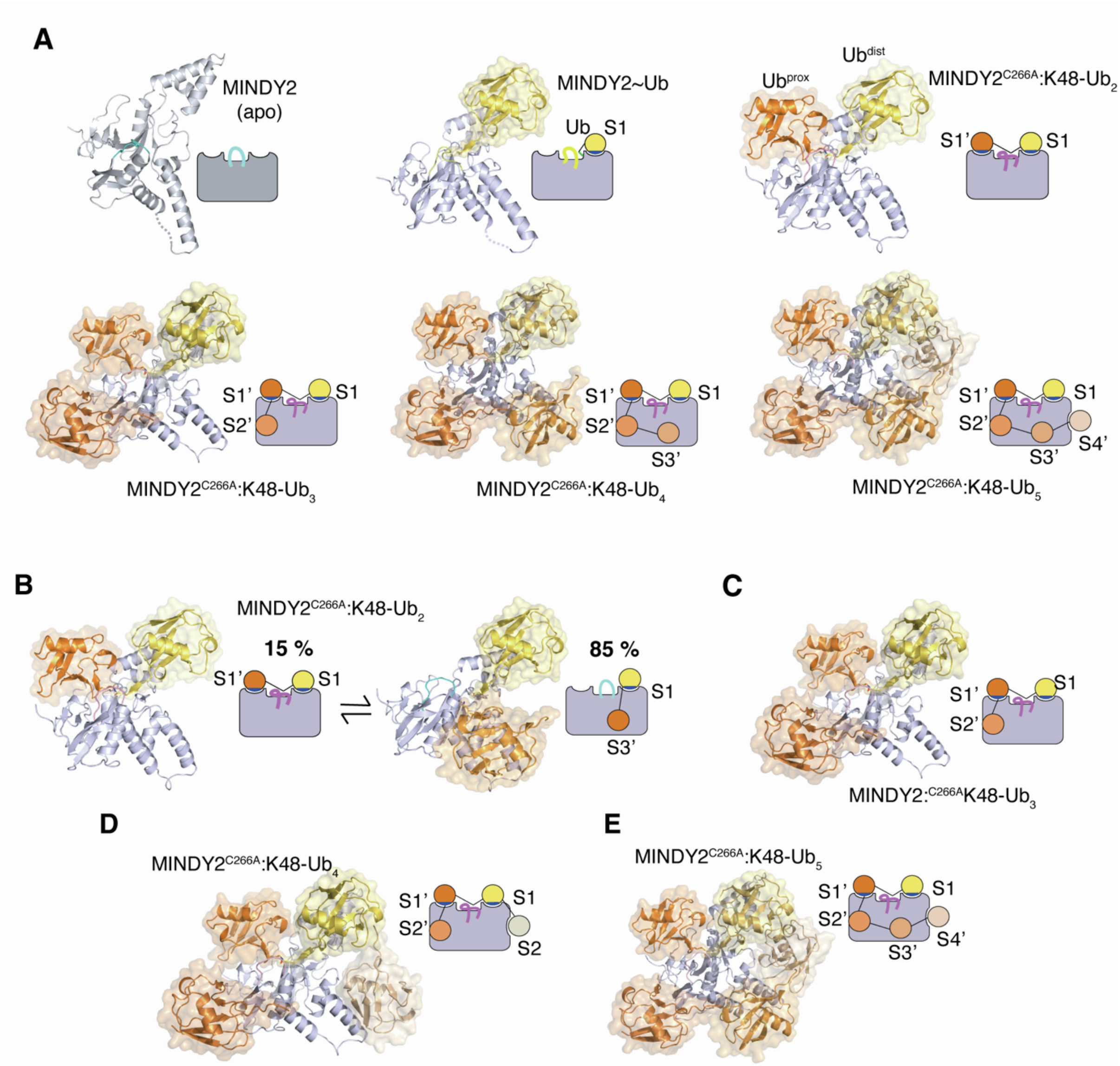

Figure S7. Small angle X-ray scattering analysis of MINDY2 complexes

A) The models used in the curve fitting with the SAXS data are depicted along with a schematic representation.

B) MINDY2^{C266A}:K48-Ub$_2$ model validated with considerable agreement.

C) MINDY2^{C266A}:K48-Ub$_3$ models observed and validated in SAXS measurements with some deviations and improved curve fitting post normal mode analysis run. The model was obtained by refinement of Ub positions.

D) MINDY2^{C266A}:K48-Ub$_4$ models were corrected after normal mode analysis and validated with considerable agreement.

E) MINDY2^{C266A}:K48-Ub$_5$ models validated with considerable agreement.

Related to Figure 5

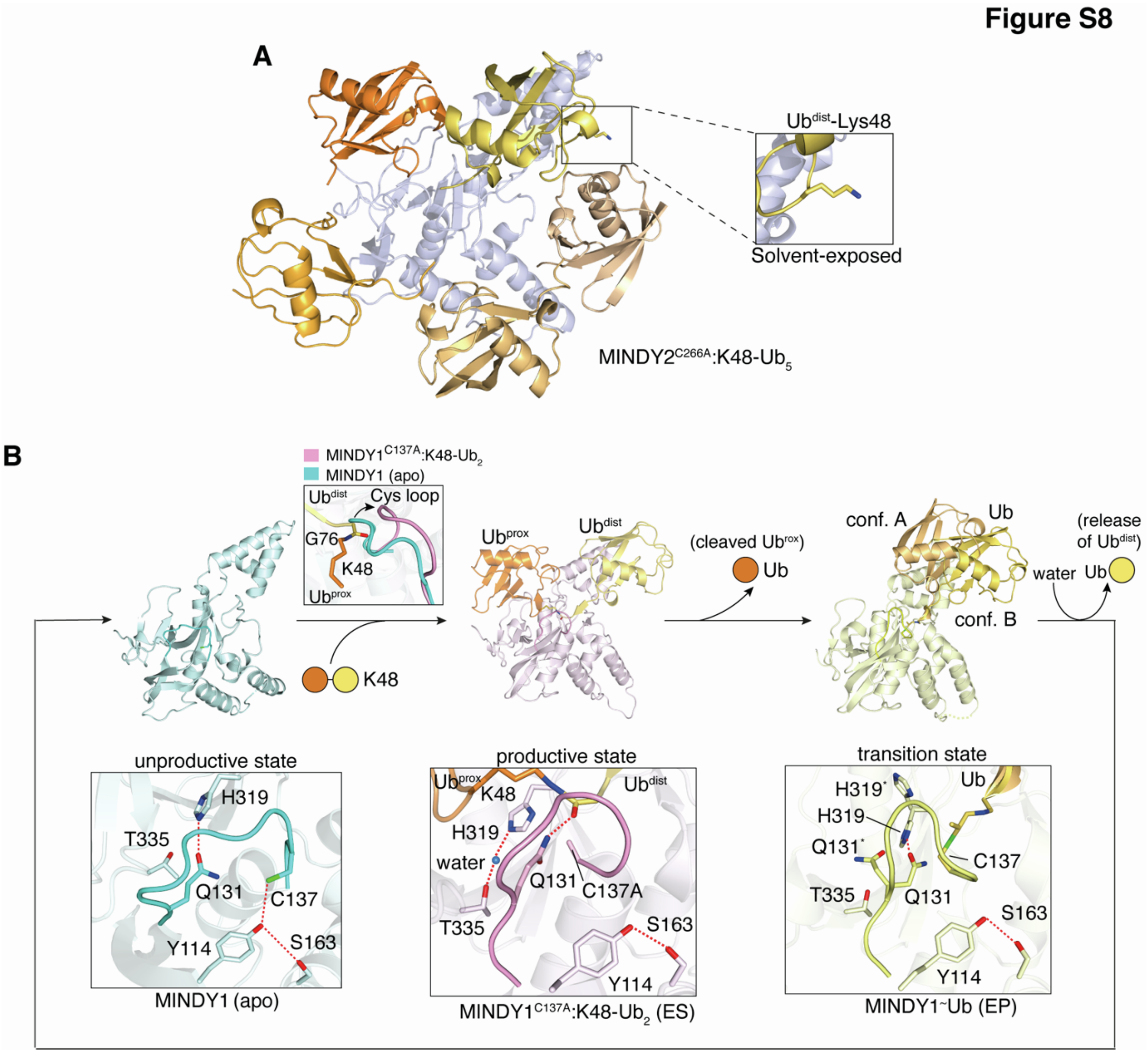

Figure S8 Mechanism of substrate recognition and cleavage by MINDY1/2

A) Crystal structure of MINDY2^{C266A}:K48-Ub5 complex highlighting the solvent-exposed Lys48 residue of Ub^{dist}.

B) Related to **Fig 7**, this shows the structures and the transitions in the catalytic cycle of MINDY1 going from inactive state to substrate bound active state and MINDY1 in the transition state bound to product intermediate.

Related to Figure 7

Table S1: Summary of major Ub interactions with DUBs, related to Figure 1

PDB ID	Title of Structure	Ub^{prox}	Ub^{dist}
6TUV, 6Z7V	MINDY1 in complex with K48-linked diubiquitin	**L 8, I44 V70, F45** R42	**L 8, I44 V70, L73** R42
6NJD	Crystal structure of RavD from Legionella pneumophila complexed with Met-1 linked di-ubiquitin	H61, K33 G35, Q2 E16, E64 D32	L8, I44, V70, L73, L73, R74 K6, R42, K48, R72
5LRV	Structure of Cezanne/OTUD7B OTU domain bound to K11-linked diubiquitin	K33, D32 E34, G35 K33, D32	I44, I36, L71, L73, Ile13, I30 L69, L8, R42, R72, R74
2ZNV	Crystal structure of human AMSH-LP DUB domain in complex with K63-linked ubiquitin dimer	F4, Gln62 E64, Asn60 K63	L73, V70, I44, L8, I36, L71 R42, R72, R74, K48, K6, His68 D39, R74
4NQL	The crystal structure of the DUB domain of AMSH orthologue, Sst2 from S. pombe, in complex with lysine 63-linked diubiquitin	F4, Gln62 E64, Q2 K63	L73, I44, V70, L8, L71, I36 L69, R42 R72, His68
5OHP	Crystal structure of USP30 (C77A) in complex with K6-linked diubiquitin	L8, F4 K6, His68 E64, K11	L71, L73, I36, Pro37, F4, G76 G75, R74, R42, R72, K48, K6 E16, F4, K6
3WXE	Crystal structure of CYLD USP domain (C596S) in complex with M1-linked diubiquitin	F4, M1 Q2, Thr12 E64, Glu18 K6, E16 F4, K63	V70, L8 I36, L69 L71, L73 G75, R74 R42, R72 Glu51, K6
3WXG	Crystal structure of CYLD USP domain (C596A) in complex with K63-linked diubiquitin	F4, E64, K63, Glu18 K29, K6, E16	V70, L8, I36, L69, L71, L73 G75, G76, R74, R42, R72, Glu51 K6
3ZNZ<br>5KSL<br>5OE7	Crystal structure of OTULIN OTU domain (C129A) in complex with M1- di ubiquitin<br>Structure of OTULIN bound to the M1-linked diubiquitin activity probe<br>Gumby/Fam105B in complex with linear di-ubiquitin	K33, D32 M1, Q2 E34, K63 K33, E34 D32, E64 K63, K29 E16, K33	L8, I44, V70 G75, G76 R42, Glu32 K11

Colour coding: Hydrophobic; Hydrogen bond; Ionic bond; Cation-Pi

Table S2: SAXS data collection, related to Figure 5

**Data collection parameters**						
Radiation source	PETRA III (DESY, Hamburg, Germany)					
Beamline	EMBL P12					
Detector	PILATUS 6M					
Beam geometry (mm, FWHM)	0.12 x 0.20					
Wavelength (nm)	0.12					
Sample-detector distance (m)	3.0					
Momentum transfer *s* range (nm^{-1})	0.01 – 7.0					
Exposure time (s)	1 s (SEC-SAXS mode)					
Temperature (°C)	20					
Buffer	20 mM Hepes, 100 mM NaCl, 5 mM DTT, pH 7.5					
Column	Superdex 200 10/300					
**Overall Parameters**						
	MINDY 2 Apo	MINDY 2 Ub 1	MINDY 2 Ub 2	MINDY 2 Ub 3	MINDY 2 Ub 4	MINDY 2 Ub 5
Number of ubiquitins	0	1	2	3	4	5
Concentration (mg/ml)	8.8	10.5	9.9	12.2	13.7	17.1
R_g from Guinier approximation (nm)	2.1±0.1	2.3±0.1	2.5±0.1	2.8±0.2	2.9±0.2	2.8±0.2
D_{max} (nm)	6.5±0.3	6.8±0.3	8.0±0.4	8.8±0.5	9.2±0.5	9.0±0.6
Excluded (Porod) volume (nm^3)	45±4	58±6	68±7	97±9	111±10	116±10
Molecular weight from Porod invariant, (kDa)	28±3	35±4	43±5	60±6	69±7	73±7
Molecular weight from sequence (kDa)	31.0	39.5	48.0	56.5	65.0	73.5
**Data analysis and modelling**						
Primary data reduction	SASFLOW					
Data processing	PRIMUS/CHROMIXS					
Calculation of theoretical data	CRYSOL					
χ^2 Crysol	2.04	1.47	5.23	2.07	3.75	1.48
Model refinement	SREFLEX					
χ^2 SREFLEX	1.49	-	-	1.15	1.32	-
RMSD	3.91	-	-	5.01	3.35	-
Multicomponent Mixture Analysis	OLIGOMER					
χ^2 OLIGOMER	-	-	1.90	-	-	-
SASBDB accession code	SASDJ93	SASDJA3	SASDJB3	SASDJC3	SASDJD3	SASDJE3

Table S3: Details of cDNA constructs used in study, related to Figures 1-6

Protein	Expressed protein	Tag Cleaved	Vector type	Plasmid	DU number
MINDY1^{FL}	GST-MINDY1 1-469	Yes	Bacterial	pGEX6P1	49563
MINDY2^{FL}	GST-MINDY2 1-621	Yes	Bacterial	pGEX6P1	46765
MINDY1^{cat}	GST-MINDY1 110-384	Yes	Bacterial	pGEX6P1	47257
MINDY2^{cat}	GST-MINDY2 241-504	Yes	Bacterial	pGEX6P1	53390
MINDY1^{cat} C137A	GST-MINDY1-C137A 110-384	Yes	Bacterial	pGEX6P1	47419
MINDY2^{cat} C266A	GST-MINDY2 C266A 241-504	Yes	Bacterial	pGEX6P1	55471
I266A	GST-MINDY1-I266A 110-384	Yes	Bacterial	pGEX6P1	47820
F339A	GST-MINDY1-F339A 110-384	Yes	Bacterial	pGEX6P1	47665
I266A-F339A	GST-MINDY1-I266A F339A 110-384	Yes	Bacterial	pGEX6P1	67842
I395A	GST-MINDY2 I395A 241-504	Yes	Bacterial	pGEX6P1	55941
F468A	GST-MINDY2 F468A 241-504	Yes	Bacterial	pGEX6P1	55878
R316A	GST-MINDY1-R316A 110-384	Yes	Bacterial	pGEX6P1	47668
N317A	GST-MINDY1-N317A 110-384	Yes	Bacterial	pGEX6P1	59172
D336A	GST-MINDY1-D336A 110-384	Yes	Bacterial	pGEX6P1	47667
P138A	GST-MINDY1-P138A 110-384	Yes	Bacterial	pGEX6P1	47661
P138G	GST-MINDY1-P138G 110-384	Yes	Bacterial	pGEX6P1	59586
P138L	GST-MINDY1-P138L 110-384	Yes	Bacterial	pGEX6P1	59683
P138W	GST-MINDY1-P138W 110-384	Yes	Bacterial	pGEX6P1	59684
P136A	GST-MINDY1-P136A 110-384	Yes	Bacterial	pGEX6P1	47821
P136A P138A	GST-MINDY1-P136A P138A 110-384	Yes	Bacterial	pGEX6P1	47815
Y114A	GST-MINDY1-Y114A 110-384	Yes	Bacterial	pGEX6P1	47659
Y114F	GST-MINDY1-Y114F 110-384	Yes	Bacterial	pGEX6P1	47671
N134A	GST-MINDY1-N134A 110-384	Yes	Bacterial	pGEX6P1	59152
S321A	GST-MINDY1-S321A 110-384	Yes	Bacterial	pGEX6P1	47670
S321D	GST-MINDY1-S321D 110-384	Yes	Bacterial	pGEX6P1	59666
T335V	GST-MINDY1-T335V 110-384	Yes	Bacterial	pGEX6P1	47757

T335D	GST-MINDY1-T335D 110-384	Yes	Bacterial	pGEX6P1	58884
P265A	GST-MINDY2 P265A 241-504	Yes	Bacterial	pGEX6P1	59611
P267A	GST-MINDY2 P267A 241-504	Yes	Bacterial	pGEX6P1	55477
P265A P267A	GST-MINDY2 P265A P267A 241-504	Yes	Bacterial	pGEX6P1	59610
P138A C137A	GST-MINDY1C137A P138A 110-384	Yes	Bacterial	pGEX6P1	55726
S450A	GST-MINDY2-S450A 241-504	Yes	Bacterial	pGEX6P1	55826
T464V	GST-MINDY2-T464V 241-504	Yes	Bacterial	pGEX6P1	55825
V242G I289A	GST-MINDY2 V242G I289A 241-504	Yes	Bacterial	pGEX6P1	70504
V242S I289A	GST-MINDY2 V242S I289A 241-504	Yes	Bacterial	pGEX6P1	70505
L304A	GST-MINDY2 L304A 241-504	Yes	Bacterial	pGEX6P1	70501
S324A D325A	GST-MINDY2 S324A D325A 241-504	Yes	Bacterial	pGEX6P1	70513
L304A S324A D325A	GST-MINDY2 L304A S324A D325A 241-504	Yes	Bacterial	pGEX6P1	70512
R342E	GST-MINDY2 R342E 241-504	Yes	Bacterial	pGEX6P1	70502
E350A	GST-MINDY2 E350A 241-504	Yes	Bacterial	pGEX6P1	55811
Y351A	GST-MINDY2 Y351A 241-504	Yes	Bacterial	pGEX6P1	70507
R343E E350A Y351A	GST-MINDY2 R343E E350A Y351A 241-504	Yes	Bacterial	pGEX6P1	70510
Ubiquitin K48R, K63C	6His-Ub K48R K63C 2-76	Yes	Bacterial	pET15b	70511
Ubiquitin 1-76	Ubiquitin (expressed tagless)	Tagless	Bacterial	pET24	20027
Ubiquitin 1-75	Ub-Intein-CBD 1-75	Yes	Bacterial	pTXB1	24149
